# Efficacy, Safety, and Biomarkers of Neoadjuvant Dalpiciclib (CDK4/6 inhibitor) plus Aromatase Inhibitors in Operable HER2‐Negative Luminal B Breast Cancer: A Prospective, Single‐Center, Single‐Arm, Phase II Trial (DANCER)

**DOI:** 10.1002/mco2.70402

**Published:** 2025-10-11

**Authors:** Yunxiang Zhou, Zhiyun Zhang, Huihui Chen, Fengbo Huang, Lu Shen, Siqi Tao, Wei Qian, Hui Hong, Chi Pan, Ze Wang, Jiao Zhang, Yue Hu, Yong Shen, Jun Fu, Weikang Mao, Shijie Wu, Xianan Guo, Hui Wang, Mindi Ma, Ting Ma, Liqiang Pan, Yiding Chen

**Affiliations:** ^1^ Department of Breast Surgery and Oncology The Second Affiliated Hospital, Zhejiang University School of Medicine Hangzhou Zhejiang China; ^2^ Cancer Institute (Key Laboratory of Cancer Prevention and Intervention, China National Ministry of Education) The Second Affiliated Hospital, Zhejiang University School of Medicine Hangzhou China; ^3^ Department of Pathology The Second Affiliated Hospital, Zhejiang University School of Medicine Hangzhou Zhejiang China; ^4^ Department of Radiology The Second Affiliated Hospital, Zhejiang University School of Medicine Hangzhou Zhejiang China; ^5^ Genecast Biotechnology Co., Ltd Wuxi Jiangsu China; ^6^ LC‐Bio Technology Co., Ltd Hangzhou China; ^7^ Department of Pathology Zhejiang Cancer Hospital Hangzhou Zhejiang China; ^8^ Department of Radiology The First Affiliated Hospital of Zhejiang Chinese Medical University (Zhejiang Provincial Hospital of Chinese Medicine) Hangzhou Zhejiang China; ^9^ Laboratory of Precision Medicine and Biopharmaceuticals College of Pharmaceutical Sciences Zhejiang University Hangzhou Zhejiang China; ^10^ Department of Pharmacy The Second Affiliated Hospital, Zhejiang University School of Medicine Hangzhou Zhejiang China

**Keywords:** luminal B breast cancer, neoadjuvant, biomarker, circulating tumor DNA, Olink proteomics, CDK4/6 inhibitor

## Abstract

Dalpiciclib, a cyclin‐dependent kinase 4/6 (CDK4/6) inhibitor, has demonstrated significant clinical activity and manageable safety in advanced luminal breast cancer, yet its neoadjuvant value remains unestablished. The single‐arm, phase II DANCER trial (NCT05640778) is the first to evaluate circulating tumor DNA (ctDNA)‐guided neoadjuvant CDK4/6 inhibitor therapy in patients with operable human epidermal growth factor receptor 2 (HER2)‐negative luminal B breast cancer, a subtype with poor chemotherapy response and unmet neoadjuvant needs. Thirty patients received dalpiciclib plus aromatase inhibitors (DAL‐AI) with ctDNA monitoring and multiomics profiling. At week 2, 26 (86.7%) patients achieved complete cell cycle arrest (primary endpoint). By surgery, 60.0% had partial response; breast pathological complete response and residual cancer burden 0–I rates were 6.7 and 3.3%, respectively. Treatment was well tolerated, with the most common grade ≥3 adverse events being neutropenia and leukopenia. Candidate predictive biomarkers included ctDNA clearance, *GSTM1* copy number, MammaPrint index, plasma CCL4/CCL19 levels, and tissue pRb/CDK4 expression. A novel baseline response index combining CCL4 and pRb showed excellent predictive performance and stratified patients by likelihood of clinical benefit, with ctDNA dynamics further refining stratification. These findings support DAL‐AI as a promising neoadjuvant option and highlight the value of biomarker‐guided strategies for treatment optimization.

## Graphical abstract

DANCER (NCT05640778) was a circulating tumor DNA (ctDNA)‐directed, single‐arm, phase II trial investigating the clinical activity of dalpiciclib combined with aromatase inhibitors as a neoadjuvant regimen for operable human epidermal growth factor receptor 2 (HER2)‐negative luminal B breast cancer. Although a high complete cell cycle arrest (CCCA) rate (primary endpoint) of 86.7% (26 out of 30) was achieved at 2 weeks, some patients showed suboptimal clinical responses after neoadjuvant therapy. Comprehensive multiomics biomarker analysis revealed that patients with early ctDNA clearance, MammaPrint low‐risk status, *GSTM1* deletion, increased tissue pRb/CDK4 expression, and higher plasma CCL4/CCL19 levels are likely to benefit more from neoadjuvant dalpiciclib therapy. The lower right (BIOMARKERS) panel was created in BioRender. Zhou, Y. (2025) https://BioRender.com/ax0bxdu.

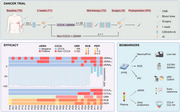

## 1 Introduction

Hormone receptor‐positive, human epidermal growth factor receptor 2‐negative (HR+/HER2−) breast cancer accounts for approximately 70% of all breast cancer cases and represents the most prevalent subtype [1]. While neoadjuvant chemotherapy remains a standard approach for locally advanced disease, it has limited efficacy in downstaging HR+/HER2− tumors and suboptimal tolerability [2]. In contrast, neoadjuvant endocrine therapy offers comparable objective response rates (ORRs) and breast‐conserving surgery rates, with a more favorable toxicity profile [3]. However, a substantial proportion of tumors exhibit intrinsic or acquired resistance to endocrine agents, resulting in inadequate treatment outcomes [4]. Therefore, strategies aimed at overcoming endocrine resistance and enhancing neoadjuvant efficacy are of considerable clinical interest.

Cyclin‐dependent kinases (CDKs) are a family of serine/threonine protein kinases that play pivotal roles in cell cycle regulation [5]. Among them, CDK4 and CDK6 are key regulators of the G1‐to‐S phase transition, and their inhibition leads to G1 arrest through suppression of retinoblastoma protein (Rb) phosphorylation [4, 5]. Combined blockade of CDK4/6 and estrogen receptor (ER) signaling has demonstrated synergistic effects, effectively delaying or reversing endocrine resistance [6]. Dalpiciclib, a selective CDK4/6 inhibitor, was approved by the China National Medical Products Administration in 2021. Large‐scale phase III trials have shown that adding dalpiciclib to endocrine therapy significantly improves outcomes in patients with both early‐ [7] and advanced‐stage [8] HR+/HER2− breast cancer. Notably, dalpiciclib plus letrozole or anastrozole achieved superior progression‐free survival and ORR compared with similar first‐line studies involving other CDK4/6 inhibitors such as abemaciclib, palbociclib, and ribociclib in advanced HR+/HER2− breast cancer [8, 9]. However, its clinical efficacy in the neoadjuvant setting remains to be established.

Several trials have evaluated the neoadjuvant use of CDK4/6 inhibitors combined with endocrine therapy, either versus chemotherapy (e.g., NeoPal [10], CORALLEEN [11], and CARABELA [12]) or versus endocrine monotherapy (e.g., PALLET [13] and neoMONARCH [4]). Despite substantial suppression of tumor proliferation, as reflected by Ki67 reduction, these studies did not show significant improvement in pathological complete response (pCR) rate, ORR, disease control rate, or preoperative endocrine prognostic index (PEPI) compared with chemotherapy or endocrine therapy alone [4, 10, 11, 12, 13, 14]. These findings underscore the need to refine patient selection, optimize treatment duration, and identify more suitable endpoints for evaluating neoadjuvant CDK4/6 inhibitor regimens.

Beyond ER positivity, robust baseline biomarkers predicting benefit from CDK4/6 inhibitors are currently lacking [4, 15]. A comprehensive multiomics profiling strategy is urgently needed to identify predictive biomarkers and resistance mechanisms. Meanwhile, dynamic pharmacodynamic markers, particularly early changes in Ki67 (e.g., complete cell cycle arrest [CCCA; Ki67 ≤ 2.7%] after 2 weeks of treatment), have been widely used to assess the efficacy and prognosis of neoadjuvant endocrine therapy [16, 17, 18, 19, 20]. Emerging evidence also suggests that circulating tumor DNA (ctDNA) may serve as a potential molecular biomarker for monitoring residual disease and prognosis after neoadjuvant therapy in breast cancer [21, 22, 23, 24], although its role in CDK4/6 inhibitor‐based neoadjuvant regimens remains unexplored. Dynamic monitoring of Ki67 and ctDNA may facilitate tailored treatment adaptation by identifying responders and redirecting nonresponders to alternative regimens.

**FIGURE 1:**
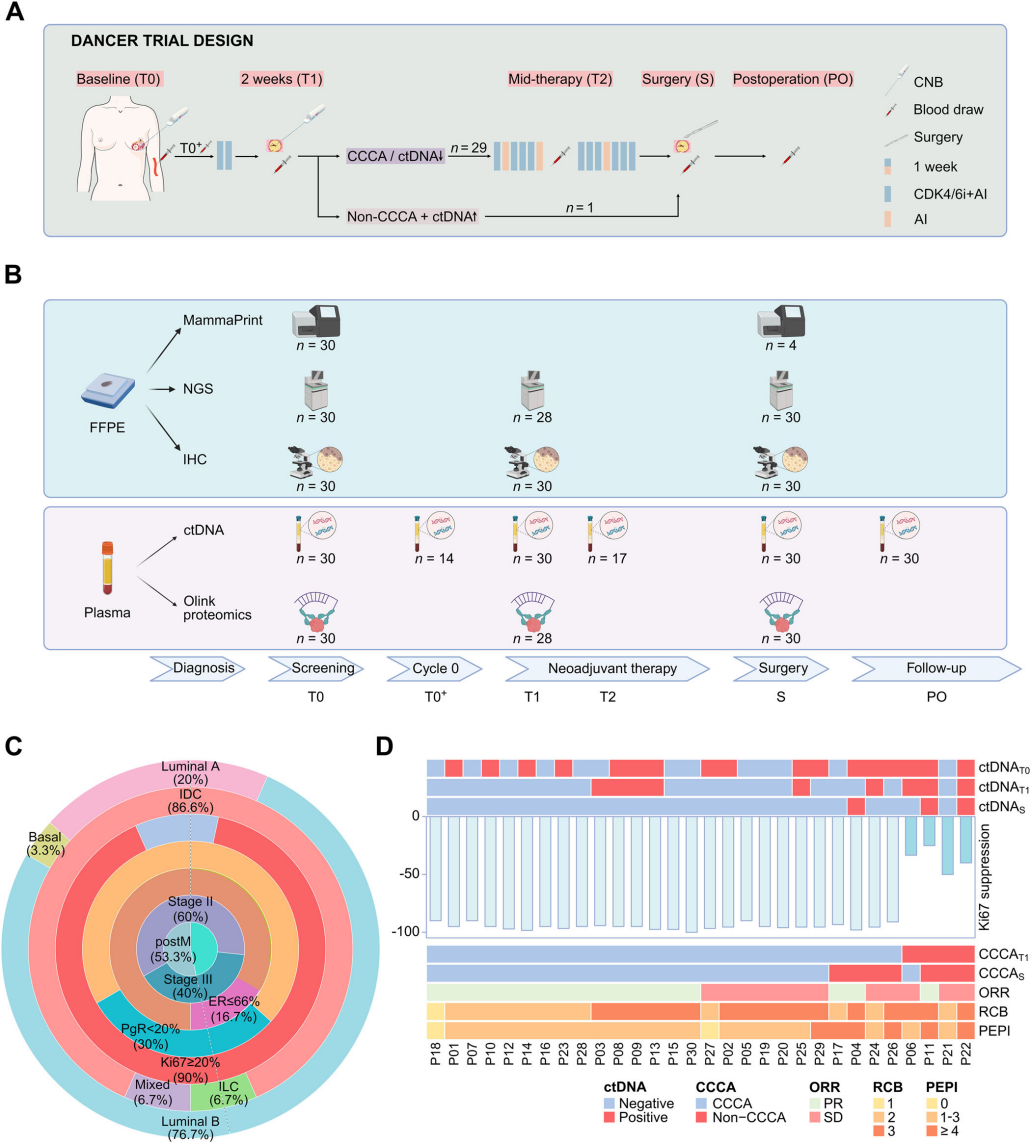
Overview of the DANCER trial. (A) Flow chart of the trial design and sample collection. T0: baseline; T0^+^: before the first dalpiciclib dose (for premenopausal patients only); T1: 2 weeks post‐dalpiciclib treatment; T2: 8 weeks post‐dalpiciclib treatment (mid‐therapy); S: surgery; PO: 2–4 weeks postoperatively. (B) Methods for biomarker analysis. Cycle 0: the 14‐day period immediately before the first dose of dalpiciclib (for premenopausal patients only). Created in BioRender. Zhou, Y. (2025) https://BioRender.com/z5yv12o. (C) Patient baseline characteristics. (D) Treatment outcomes. Ki67 suppression was calculated as [(Ki67_T1_/Ki67_T0_) − 1] × 100%. *Abbreviations*: CCCA, complete cell cycle arrest; ctDNA, circulating tumor DNA; CNB, core needle biopsy; CDK4/6, cyclin‐dependent kinase 4/6; FFPE, formalin‐fixed and paraffin‐embedded; NGS, next‐generation sequencing; IHC, immunohistochemistry; IDC, invasive ductal carcinoma; ILC, invasive lobular carcinoma; postM, postmenopausal; ER, estrogen receptor; PgR, progesterone receptor; ORR, overall response rate; RCB, residual cancer burden; PEPI, preoperative endocrine therapy prognosis index; PR, partial response; SD, stable disease.

HER2‐negative luminal B breast cancer is a biologically aggressive HR+/HER2− subtype characterized by lower progesterone receptor (PgR) expression, higher Ki67 levels, and worse clinical outcomes [25]. These tumors are more likely to exhibit aberrant activation of the cyclin D–CDK4/6–INK4–Rb pathway, potentially rendering them more sensitive to CDK4/6 inhibitors [6, 25]. Herein, we conducted the DANCER study to evaluate the efficacy, safety, and predictive biomarkers of dalpiciclib plus aromatase inhibitor (DAL‐AI) as neoadjuvant therapy for HER2‐negative luminal B breast cancer (Figure 1A). The trial employed a response‐guided adaptive design, in which patients who failed to achieve CCCA and simultaneously exhibited ctDNA elevation at week 2 of DAL‐AI therapy (T1) were reassigned to alternative therapy. Others continued DAL‐AI for a total of 15 weeks prior to surgery. Plasma samples were collected at baseline (T0), T1, mid‐therapy (T2), surgery (S), and postoperatively (PO) for ctDNA and Olink proteomic analyses. Tumor tissues obtained at T0, T1, and S were subjected to somatic mutation profiling, immunohistochemical analysis, and MammaPrint test (Figure 1B). By integrating multiomics data, we aim to delineate optimal responders to CDK4/6 inhibition and facilitate future refinement of neoadjuvant strategies for this challenging subtype.

## 2 Results

### 2.1 Baseline Characteristics

Between October 28, 2022 and June 26, 2024, a total of 40 patients underwent eligibility screening for enrollment. Seven patients were found to be ineligible, and two withdrew their informed consent prior to treatment initiation. Ultimately, 31 patients received DAL‐AI therapy, while one patient was subsequently excluded from the final analysis after bilateral breast cancer was reconfirmed by pathological biopsy during surgery. The baseline characteristics of the included 30 patients are presented in Table 1 and Figure 1C. The median age of the patients was 52.5 years (range, 38.0–76.0 years), with an equal distribution between postmenopausal (*n* = 16) and premenopausal (*n* = 14) women. The analyzed patients had a relatively high tumor burden, with a median tumor size of 33.5 mm (range, 21.3–98.9 mm) assessed by magnetic resonance imaging (MRI), and the majority (25 out of 30, 83.3%) had lymph node involvement. Twenty‐five (83.3%) patients had an ER expression level of >66%, and 27 (90.0%) had a Ki67 level of ≥20%. According to MammaPrint and BluePrint assays, 80.0% (24 out of 30) of the tumors were classified as genomic high‐risk; one patient had Basal‐type disease, while the other 29 had luminal‐type disease.

**TABLE 1:** Patient baseline characteristics.

Characteristic	*n* (%)
Age (years), median (range)	52.5 (38.0–76.0)
Menstrual status	
Premenopausal	14 (46.7)
Postmenopausal	16 (53.3)
Clinical tumor stage	
T2	18 (60.0)
T3/4	12 (40.0)
Clinical nodal stage	
N0	5 (16.7)
N1	19 (63.3)
N2	6 (20.0)
Clinical tumor stage	
II	18 (60.0)
III	12 (40.0)
Histological type	
IDC	26 (86.7)
ILC	2 (6.7)
Mixed^a^	2 (6.7)
Histological tumor grade	
1–2	28 (93.3)
3	2 (6.7)
ER expression	
11–66%	5 (16.7)
>66%	25 (83.3)
PgR expression	
<1%	4 (13.3)
≥1%, <20%	5 (16.7)
≥20%	21 (70.0)
Ki67 expression	
<20%	3 (10.0)
≥20%	27 (90.0)
MammaPrint/BluePrint signature	
Luminal A	6 (20.0)
Luminal B	23 (76.7)
Basal	1 (3.3)

^a^ Including two cases of mixed carcinoma, each with 10–90% lobular or micropapillary subtype admixed with invasive ductal carcinoma (IDC).

*Abbreviations*: ER, estrogen receptor; ILC, invasive lobular carcinoma; PgR, progesterone receptor.

### 2.2 Efficacy

All patients exhibited Ki67 suppression at T1, with a median Ki67 expression of 1.0% (interquartile range [IQR], 1.0–2.0%), representing a sharp decrease from the median Ki67 value of 27.5% (IQR, 20.0–40.0%) at T0 (*p* < 0.001). Overall, 86.7% (95% confidence interval [CI], 69.3–96.2%; *n *= 26) of the tumors demonstrated CCCA (Table 2 and Figure 1D), while Ki67 expression shifted from ≤2.7% at T1 to >2.7% at S in four (four out of 26, 15.4%). In contrast, one (one out of four, 25.0%) patient whose tumor did not achieve CCCA at T1 had a continued decline in Ki67 levels to 2.0% at S.

**TABLE 2:** Pathological and clinical responses.

	*n* (%)	95% CI
CCCA at T1 (primary endpoint)	26 (86.7)	69.3–96.2
CCCA at S	23 (76.7)	57.7–90.1
ctDNA		
Positive at T1	9 (30.0%)	14.7–49.4
Positive at S	3 (10.0%)	2.1–26.5
RCB		
0–1	1 (3.3)	0.1–17.2
2–3	29 (96.7)	82.8–99.9
Miller–Payne grading		
1–2	11 (36.7)	19.9–56.1
3–5	19 (63.3)	43.9–80.1
bpCR	2 (6.7)	0.8–22.1
tpCR	0 (0)	0–11.6
PEPI score (BCSS)		
0	2 (6.7)	0.8–22.1
1–3	22 (73.3)	54.1–87.7
≥4	6 (20.0)	7.7–38.6
PEPI score (RFS)		
0	2 (6.7)	0.8–22.1
1–3	22 (73.3)	54.1–87.7
≥4	6 (20.0)	7.7–38.6
Overall response by MRI		
Complete response	0 (0)	0–11.6
Partial response	18 (60.0)	40.6–77.3
Stable disease	12 (40.0)	22.7–59.4
Progression disease	0 (0)	0–11.6
Overall response by palpation		
Complete response	6 (20.0)	7.7–38.6
Partial response	19 (63.3)	43.9–80.1
Stable disease	5 (16.7)	5.6–34.7
Progression disease	0 (0)	0–11.6

*Abbreviations*: BCSS, breast cancer‐specific survival; bpCR, breast pathological complete response; CCCA, complete cell cycle arrest; CI, confidence interval; ctDNA, circulating tumor DNA; MRI, magnetic resonance imaging; PEPI, preoperative endocrine therapy prognosis index; RCB, residual cancer burden; RFS, relapse‐free survival; tpCR, total pathological complete response.

**FIGURE 2:**
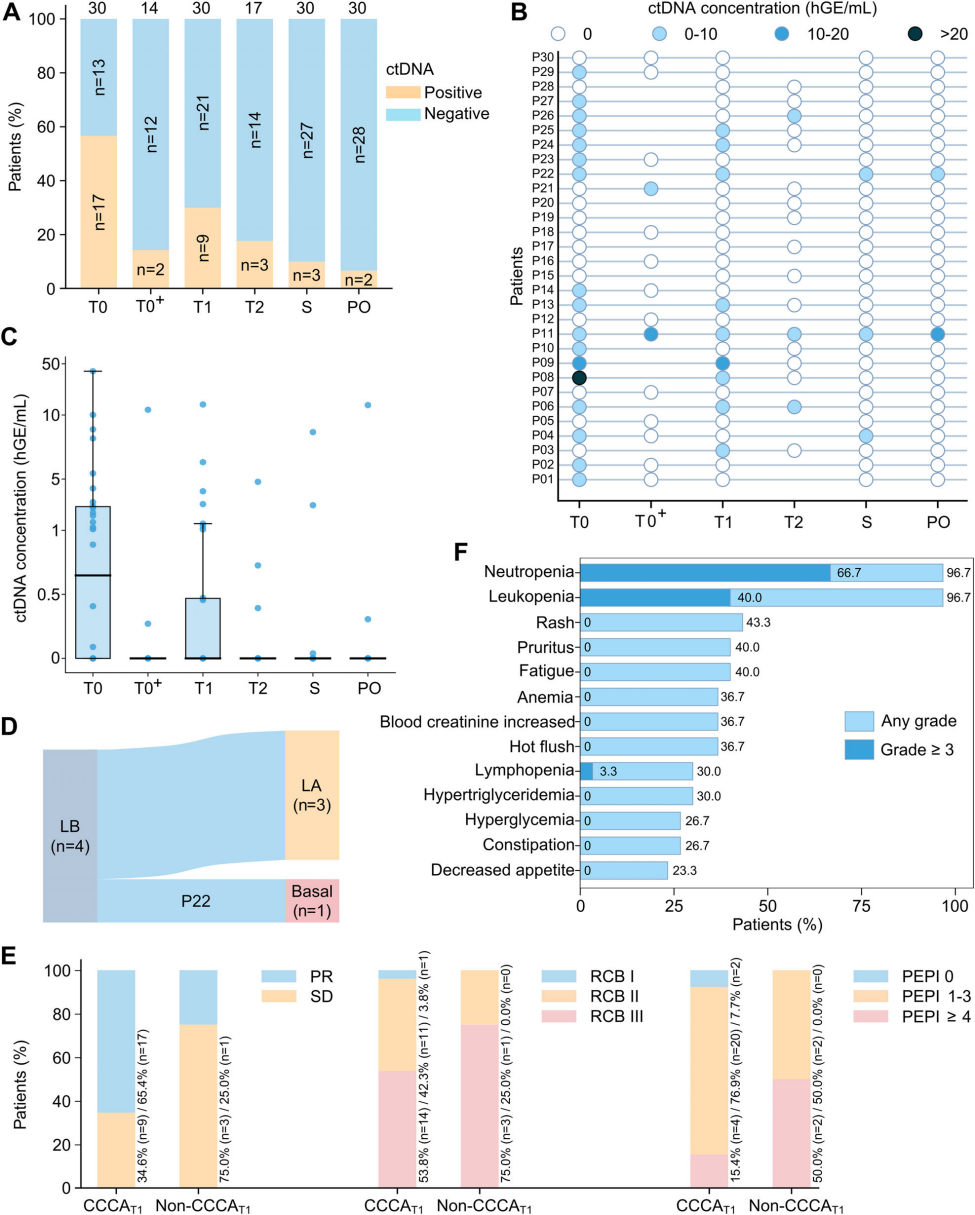
Circulating tumor DNA (ctDNA) dynamics, treatment response, and safety in patients receiving dalpiciclib. (A) Proportion of patients with ctDNA positivity based on available sample numbers at each time point. (B) Individual ctDNA trajectories of all 30 patients during neoadjuvant therapy. (C) ctDNA concentration (hGE/mL) across different time points. All bar values are presented as medians ± interquartile ranges. (D) Changes in intrinsic molecular subtypes before and after neoadjuvant therapy in patients with Miller–Payne (MP) grade 1 (*n* = 4, including Patient #22 who discontinued dalpiciclib and underwent immediate surgery). (E) Proportion of patients with different radiological and pathological responses, grouped by cell cycle arrest status 2 weeks post‐dalpiciclib treatment (T1). (F) Treatment‐emergent adverse events with an incidence greater than 20%. T0: baseline; T0^+^: before the first dalpiciclib dose, only for premenopausal patients; T2: 8 weeks post‐dalpiciclib treatment (mid‐therapy); S: surgery; PO: 2–4 weeks postoperatively. *Abbreviations*: LA, luminal A; LB, luminal B; PR, partial response; SD, stable disease; RCB, residual cancer burden; PEPI, preoperative endocrine therapy prognosis index; CCCA, complete cell cycle arrest.

A total of 151 plasma samples from the 30 patients underwent ctDNA sequencing analysis. At T0, ctDNA was detected in 17 patients. Both the positivity rate and concentration of ctDNA decreased over time during the course of DAL‐AI therapy in the entire patient population (Figure 2A–C). However, ctDNA concentrations increased in eight patients during treatment, with four experiencing an increase at T1 (Figure 2B). Specifically, one (Patient #22) of these four patients concurrently had a non‐CCCA tumor and, in accordance with the trial design and patient preference, subsequently discontinued DAL‐AI treatment and proceeded directly to surgical intervention. The remaining 29 patients completed four cycles of DAL‐AI therapy before surgery (Figure 1A). Premenopausal patients with detectable ctDNA at baseline exhibited a high ctDNA clearance rate of 85.7% (six out of seven) at the second analysis (T0^+^). In contrast, postmenopausal patients with baseline detectable ctDNA had a clearance rate of only 30% (three out of 10) at the second analysis (T1). This difference may be attributed to the flare‐up effect of gonadotropin‐releasing hormone (GnRH) agonist administration in premenopausal patients on the estrogen response, which may inhibit the shedding of ctDNA [21]. Three patients did not clear ctDNA at S, and two of them still had detectable ctDNA at PO.

The proportion of patients at surgery with breast pCR (bpCR) was 6.7% (95% CI, 0.8–22.1%; *n *= 2), but no patients achieved total pCR (tpCR). The residual cancer burden (RCB) 0–I rate was 3.3% (95% CI, 0.1–17.2%; *n *= 1). The pathological regression in four (13.3%) patients reached only a Miller–Payne (MP) grade of 1. Further multigene assays conducted on their surgically resected tumor tissues revealed that, apart from Patient #22, whose intrinsic molecular subtype transitioned from luminal B to basal‐type, the intrinsic subtypes of the remaining three patients achieved molecular downstaging from luminal B to luminal A (Figure 2D). For both breast cancer‐specific survival (BCSS) and relapse‐free survival (RFS) PEPI scores, the number of patients at low, intermediate, and high risk of relapse was 2 (6.7%), 22 (73.3%), and 6 (20.0%), respectively.

MRI measurements showed that, at T2, 14 (48.3%; 95% CI, 29.4–67.5%) out of the 29 patients continuing DAL‐AI therapy achieved a partial response (PR). By S, the ORR had increased to 60.0% (95% CI, 40.6–77.3%), with 18 out of 30 tumors demonstrating PR. When assessed by physical examination, the ORR reached 83.3%. The patients who achieved CCCA at T1 tended to exhibit better radiological and pathological responses (Figures 2E and S1). Among the four tumors that failed to exhibit CCCA at T1, three were classified as stable disease by MRI at S, while the remaining one showed a marginal PR with a barely adequate reduction in tumor size of 31.1%.

### 2.3 Safety

All patients experienced at least two treatment‐emergent adverse events (TEAEs), with most being grade 1 (Table S1). The most common TEAEs of any grade were neutropenia (29, 96.7%), leukopenia (29, 96.7%), rash (13, 43.3%), pruritus (12, 40.0%), and fatigue (12, 40.0%) (Figure 2F). Over 30% of the patients experienced anemia, increased blood creatinine, and hot flushes. Twenty (66.7%) patients had grade ≥3 TEAEs, which resulted in no clinical consequence with symptomatic treatment. Among these, the most frequent was neutropenia (grade 3: 16, 53.3%; grade 4: 4, 13.3%), followed by leukopenia (grade 3: 11, 36.7%; grade 4: 1, 3.3%), lymphopenia (grade 3: 1, 3.3%), and stomatitis (grade 3: 1, 3.3%). However, no clinical sequelae occurred. No cases of febrile neutropenia or grade 5 adverse events were recorded.

A total of 10 patients required dalpiciclib dose reductions due to recurrent grade ≥3 neutropenia, with all such episodes first occurring during the first cycle (Table S2). Specifically, five patients experienced a decrease in neutrophil counts, with or without concurrent leukopenia, within 1 week after DAL‐AI administration. Among these five patients, four subsequently experienced recurrent grade 3 or 4 neutropenia, necessitating dalpiciclib dose adjustments. Additionally, 12 patients had grade ≥3 neutropenia within 2 weeks, with eight continuing to experience episodes. Only one patient who required a dose reduction first experienced grade 3 neutropenia at the end of Cycle 1. No patients permanently discontinued dalpiciclib. These data suggest that patients who exhibit early hematological toxicity are more prone to experiencing recurrent neutropenia.

### 2.4 Biomarker Analysis

**FIGURE 3:**
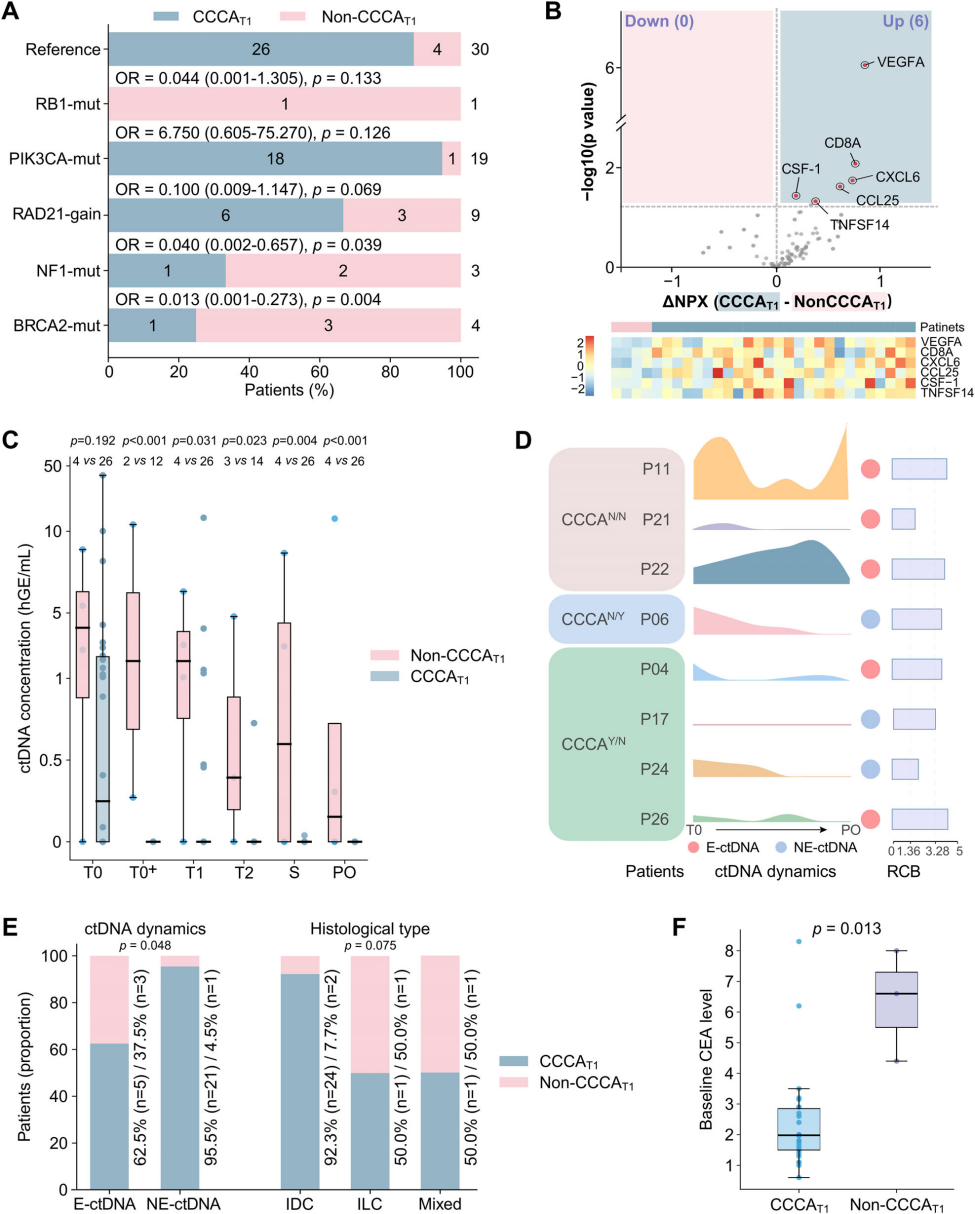
Biomarker analysis of complete cell cycle arrest (CCCA) at 2 weeks post‐dalpiciclib (T1). (A) Association of baseline (T0) genetic variations with CCCA. (B) Comparison of baseline plasma protein levels between patients with CCCA (*n* = 26) and without CCCA (*n* = 4), presented in a volcano plot (top) and a heatmap (bottom). (C) Circulating tumor DNA (ctDNA) concentration (hGE/mL) over time by cell cycle arrest status. (D) ctDNA dynamics in patients without CCCA at T1 or surgery (S) (*n* = 8). CCCA^N/N^: no CCCA at either T1 or S; CCCA^N/Y^: CCCA only at S; CCCA^Y/N^: CCCA only at T1. E‐ctDNA: ctDNA increased during neoadjuvant therapy; NE‐ctDNA: no increase. (E) CCCA proportion by ctDNA dynamics and tumor histological type. (F) Baseline serum carcinoembryonic antigen (CEA) levels grouped according to cell cycle arrest status. All bar values are presented as medians ± interquartile ranges. T0^+^: before the first dalpiciclib dose, only for premenopausal patients; T2: 8 weeks post‐dalpiciclib treatment (mid‐therapy); PO: 2–4 weeks postoperatively. *Abbreviations*: Mut, mutation; NPX, normalized protein expression; RCB, residual cancer burden; IDC, invasive ductal carcinoma; ILC, invasive lobular carcinoma.

At T1, only four patients had not achieved CCCA, making the subgroup too small for a formal statistical analysis. However, we identified certain potential baseline biomarkers associated with CCCA. Specifically, the mutation rates of *NF1* and *BRCA2* in patients with CCCA (one out of 26, 3.8%; and one out of 26, 3.8%, respectively) were significantly lower than those in patients without CCCA (two out of four, 50.0%; and three out of four, 75.0%, respectively), with *p* values of 0.039 and 0.004, respectively (Figure 3A). Additionally, baseline levels of plasma proteins, such as VEGFA, were significantly higher in patients who achieved CCCA (Figure 3B). CCCA and ctDNA are inextricably intertwined. Patients who failed to achieve CCCA at T1 exhibited significantly higher ctDNA levels during and after DAL‐AI therapy, but not at baseline, compared with those who achieved CCCA (Figure 3C). Among the four patients without CCCA at T1, three showed an increase in ctDNA during treatment, and two of them remained ctDNA‐positive until PO. Furthermore, two of the four patients who achieved CCCA at T1 but subsequently developed non‐CCCA at S had a recurrence of ctDNA positivity after an initial clearance (Figure 3D). A significant correlation between CCCA status at T1 and the dynamic trend in ctDNA was found in all 30 patients (Figure 3E). Additionally, baseline carcinoembryonic antigen (CEA) levels were significantly associated with CCCA at T1 (Figure 3F), and histological grade showed a trend toward association (*p* = 0.075; Figure 3E). However, these findings were constrained by the limited sample size.

Despite a high CCCA rate (primary endpoint) of 86.7% at T1 in this study, a certain proportion of these patients exhibited suboptimal imageological and pathological responses at the completion of neoadjuvant therapy. Thus, we stratified patients based on proliferation inhibition reflected by Ki67 levels and tumor shrinkage evaluated by MRI to identify tumors that are more likely to exhibit an optimal response to DAL‐AI. Accordingly, patients who achieved CCCA at both T1 and S, and concurrently attained a PR at S, were classified as good responders (GRs; *n* = 15). Otherwise, patients were designated as moderate responders (MRs; *n* = 15).

**FIGURE 4:**
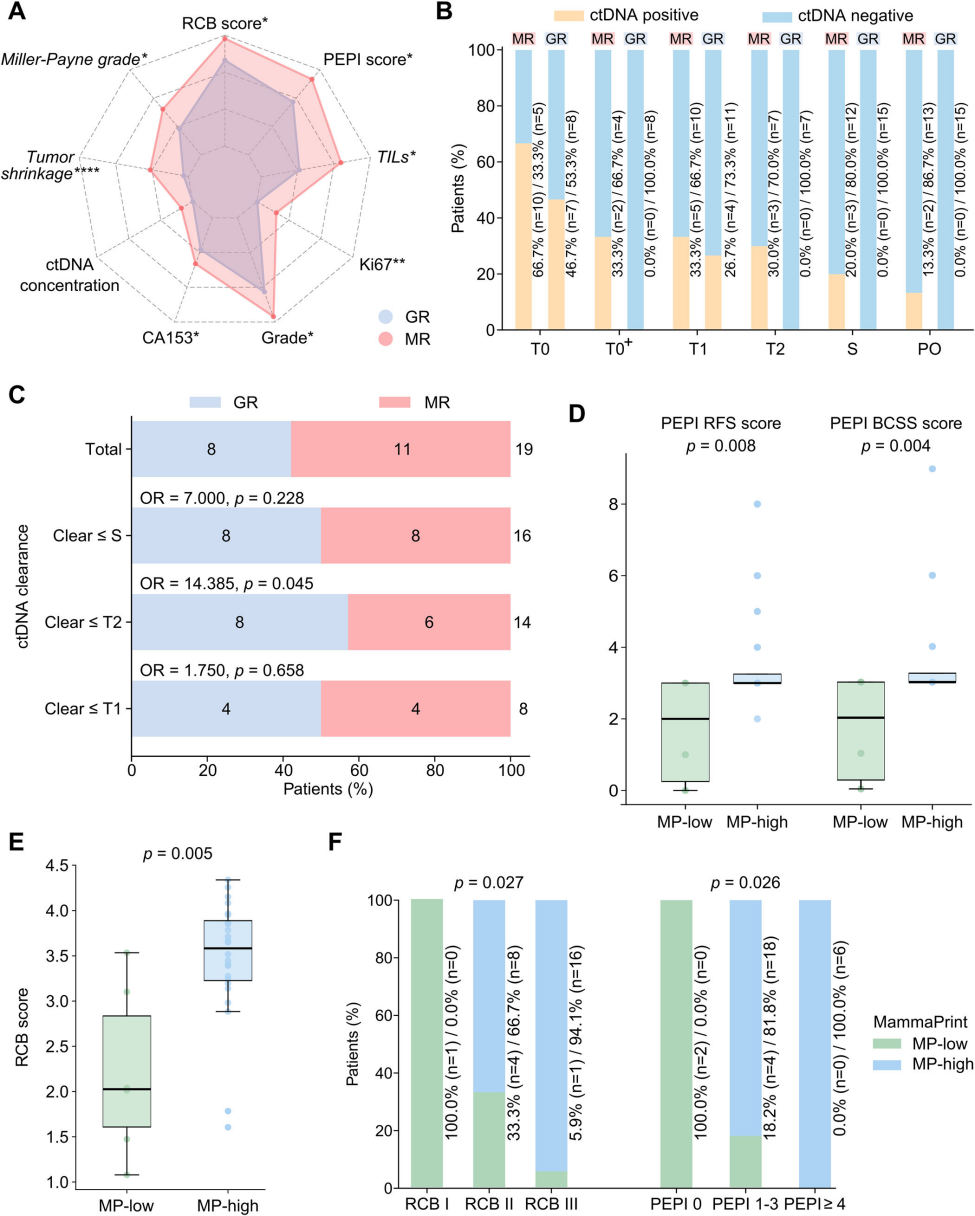
Circulating tumor DNA (ctDNA) and MammaPrint as predictive biomarkers for dalpiciclib response. (A) Radar plot comparing the efficacy and prognostic parameters at surgery (S) between good responders (GRs, *n* = 15) and moderate responders (MRs, *n* = 15). Miller–Payne grade, tumor shrinkage rate, and tumor‐infiltrating lymphocyte (TIL) level were italicized to indicate that their raw values were transformed using reciprocal (1/*x*) conversion prior to analysis. (B) Proportion of patients with ctDNA positivity based on available sample numbers per time point, grouped by GR and MR. T0: baseline; T0^+^: before the first dalpiciclib dose, only for premenopausal patients; T1: 2 weeks post‐dalpiciclib treatment; T2: 8 weeks post‐dalpiciclib treatment (mid‐therapy); PO: 2–4 weeks postoperatively. (C) Association between ctDNA clearance and GR. Clear ≤ T1: ctDNA clearance at T0^+^ or T1, without rebound; clear ≤ T2: ctDNA clearance at T0^+^, T1, or T2, without rebound; clear ≤ S: ctDNA clearance at T0^+^, T1, T2, or S, without rebound. (D and E) MammaPrint high‐risk patients exhibited higher preoperative endocrine therapy prognosis index (PEPI) (D) and residual cancer burden (RCB) (E) scores compared with low‐risk patients. (F) MammaPrint high‐risk proportion by RCB class and PEPI risk group. All bar values are presented as medians ± interquartile ranges. *p < 0.05, **p < 0.01, ****p < 0.0001. *Abbreviations*: CA153, cancer antigen 153; OR, odds ratio; RFS, relapse‐free survival; BCSS, breast cancer‐specific survival; MP‐low, MammaPrint low risk; MP‐high, MammaPrint high risk.

The baseline clinicopathological features of the tumors were balanced between the GR and MR groups (Table S3). At S, GRs exhibited significantly lower RCB scores, PEPI scores, histological tumor grades, Ki67 expression levels, and CA153 levels than MRs, and nominally lower ctDNA concentrations were also observed (Figure 4A). Additionally, GRs demonstrated significantly higher MP grades, tumor‐infiltrating lymphocyte (TIL) levels, and greater tumor shrinkage rates. These data suggest that such grouping is appropriate and holds significant clinical relevance, facilitating the exploration of genomic and proteomic biomarkers in DAL‐AI treatment.

#### 2.4.1 Circulating Tumor DNA

While ctDNA was detectable in some MRs at T0^+^, T2, S, and PO, it was consistently undetectable in all GRs at the same time points, although the difference did not reach statistical significance (Figure 4B). We further assessed ctDNA clearance as a predictive marker for DAL‐AI response. Among the 19 patients with detectable ctDNA, five (26.3%) remained ctDNA positive at T2 and/or S. The proportion of MR was significantly higher among these patients compared with the patients who cleared ctDNA at and prior to T2 (100.0% [five out of five] vs. 42.8% [six out of 14]; odds ratio [OR] = 14.385; *p* = 0.045; Figure 4C). Additionally, a higher proportion of patients who remained ctDNA‐positive until T2/S had a PEPI score of ≥4 compared with those who cleared ctDNA at or before T2 (80.0% [four out of five] vs. 7.1% [one out of 14]; OR = 52; *p *= 0.006; Figure S2A,C). Similarly, RCB class of III was more frequent in patients who remained positive for ctDNA until T1/T2/S than in those who cleared ctDNA at or before T1 (90.9% [10 out of 11] vs. 25.0% [two out of eight]; OR = 30; *p* = 0.006; Figure S2B,D).

#### 2.4.2 MammaPrint

Although no difference in MammaPrint risk groups was observed between GRs and MRs, we found that both BCSS and RFS PEPI scores, as well as RCB scores, were significantly higher in MammaPrint high‐risk patients compared with MammaPrint low‐risk patients (Figure 4D,E). Furthermore, MammaPrint high‐risk tumors were significantly more frequent in patients with a higher RCB class or a higher PEPI risk group (Figure 4F). Interestingly, patients with higher MammaPrint risk scores were more inclined to achieve pCR when undergoing neoadjuvant chemotherapy [26, 27, 28]. These contrasting findings may indeed reflect the distinct patient populations benefiting from each therapy.

#### 2.4.3 Genovariation

**FIGURE 5:**
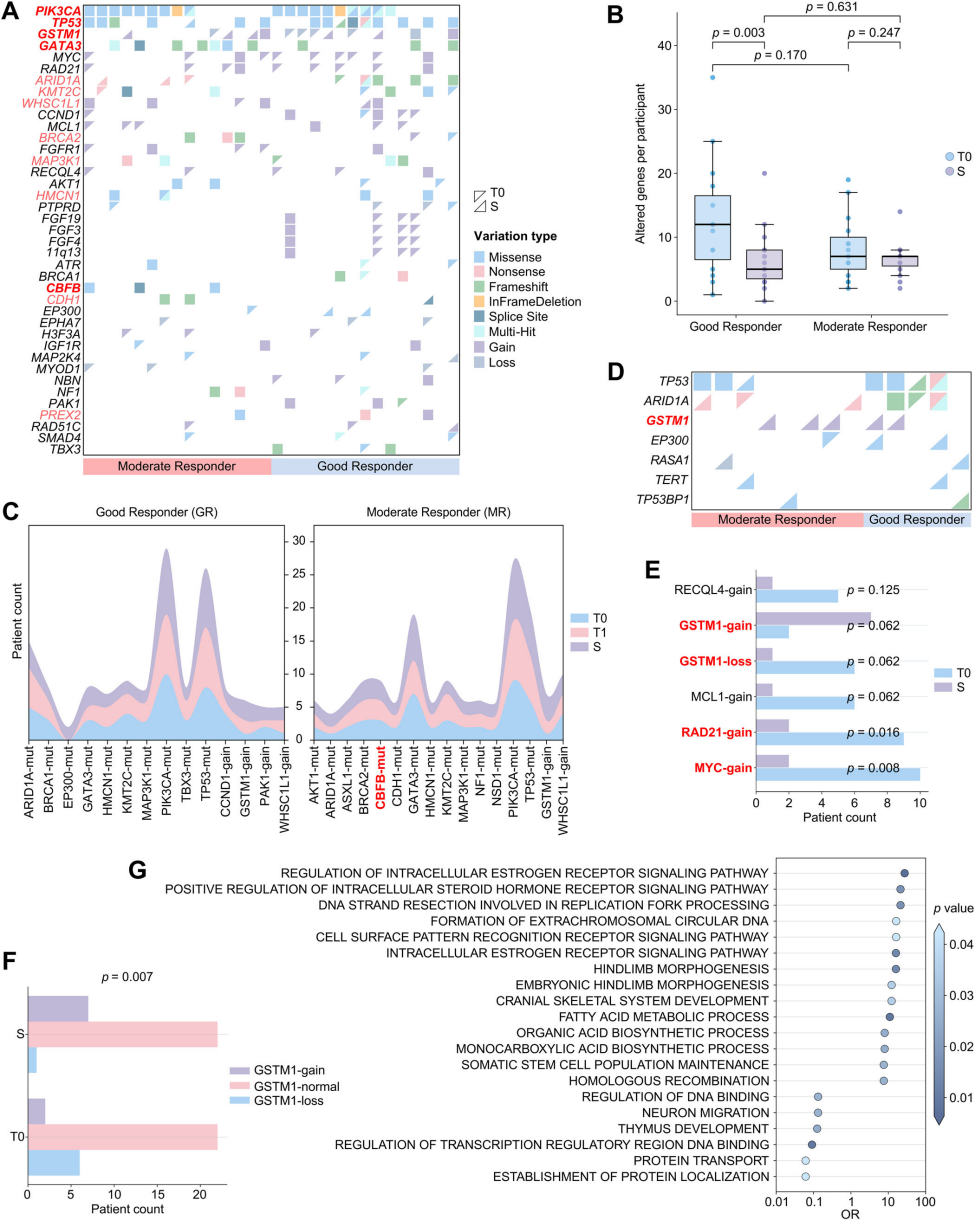
Genetic alterations and variant dynamics in good responders (GRs) and moderate responders (MRs) to dalpiciclib therapy. (A) Landscape of high‐frequency altered genes (≥10%) at baseline (T0) and surgery (S) in GRs (*n* = 15) and MRs (*n* = 15). Genes with consistently high variation frequency at S are labeled in red font, with key genes of interest further emphasized in bold red. (B) Change in variation counts after dalpiciclib therapy in patients with different responses. All bar values are presented as medians ± interquartile ranges. (C) Temporal changes in genetic variations at T0, 2 weeks post‐dalpiciclib treatment (T1), and S in GRs and MRs, showing genes with a variation frequency ≥13.3% (two out of 15) at S. (D) Landscape of de novo variations in seven genes with newly emerged variants in ≥2 patients at S. (E) Patterns of variant frequency changes after dalpiciclib therapy. Only genetic variants with *p* < 0.2 are shown. (F) Significant difference in *GSTM1* copy number after dalpiciclib therapy. The copy number of *GSTM1* was categorized into deletion (loss), normal, and amplification (gain). (G) Analysis of alterations in gene sets derived from the Gene Ontology Biological Process ontology between GRs and MRs at T0. *Abbreviation*: OR, odds ratio.

The landscape of high‐frequency altered genes (≥10%) in GRs and MRs is presented in Figure 5A. Among the 30 tissues obtained at T0, each harbored at least one somatic genetic variation in cancer‐related genes, with a median of eight variations (range, 1–35). DAL‐AI treatment significantly reduced the number of variations at S in GRs (*p* = 0.003; Figure 5B) as well as the overall population (*p* = 0.001; Figure S3A). The most frequently mutated genes at T0 were *PIK3CA* (19 out of 30, 63.3%), *TP53* (14 out of 30, 46.7%), and *GATA3* (10 out of 30, 33.3%), and all patients carrying these mutations were found to still harbor them at S. Certain genetic alterations demonstrated a preference between the response status groups. For instance, amplifications on chromosome 11q13, involving FGF family genes (*FGF3*, *FGF4*, and *FGF19*), were exclusively observed in GRs at T0 (four out of 15, 26.7%), with a remarkable decline in frequency posttreatment at S. Notably, mutations in *CBFB* (which encodes a transcription factor pivotal for suppressing breast cancer [29]; three out of 15, 20.0%) were enriched in MRs but were not detected in GRs, and this distinction persisted at T1 and S, suggesting a potential role in DAL‐AI resistance (Figure 5C). Furthermore, the presence of wild‐type *CBFB* could serve as a significant predictor of sustained CCCA and concurrent ctDNA clearance at S (Figure S3B).

Thirteen genes with variation frequencies ≥10% at S were found in the overall population (Figure 5A). Additionally, seven genes exhibited de novo variations in two to five patients at S, with *GSTM1* showing the most (Figure 5D). *GSTM1* deletion, which was numerically more common in GRs than MRs (33.3 vs. 6.7%; *p *= 0.169) at T0, nominally decreased after treatment, whereas its amplification nominally increased (Figure 5E). When the copy number was categorized into deletion, normal, and amplification, a significant increase in *GSTM1* copy number was revealed after DAL‐AI treatment (*p* = 0.007; McNemar–Bowker test; Figures 5F and S3C). Moreover, the frequencies of *MYC* and *RAD21* amplifications significantly decreased at S, while no significant differences in the frequencies of single nucleotide variants (SNVs) were observed after DAL‐AI treatment. The pattern of variant frequency changes did not differ between GRs and MRs (Figure S3D).

We conducted an analysis of gene set alterations between GRs and MRs to identify pathways associated with response to DAL‐AI. At T0, the alteration frequencies in 26 Gene Ontology gene sets (Molecular Signatures Database [MSigDB], v2024.1.Hs) significantly differed between the response groups. Among these, 14 Biological Process (BP) gene sets, including those related to ER signaling and DNA repair, exhibited a significantly higher frequency of alterations in GRs compared with MRs (Figure 5G and Table S4). In contrast, six gene sets, including those involved in transcription and protein transport, were more frequently altered in MRs. A longitudinal analysis revealed that GRs exhibited more altered BP gene sets at T1 (Figure S4A), potentially reflecting their early response to DAL‐AI. By S, this pattern shifted, with MRs showing greater relative accumulation of alterations (Figure S4B), possibly indicative of more effective clonal clearance in GRs. Notably, the gene set *activation of innate immune response* was nominally more frequently altered in GRs at T0 (*p* = 0.065), with verifiable activating events (amplifications and activating mutations) observed (Table S5), while alterations in the gene set innate immune response activating cell surface receptor signaling pathway were significantly more frequent in GRs than in MRs at S.

#### 2.4.4 Peripheral Immunome

**FIGURE 6:**
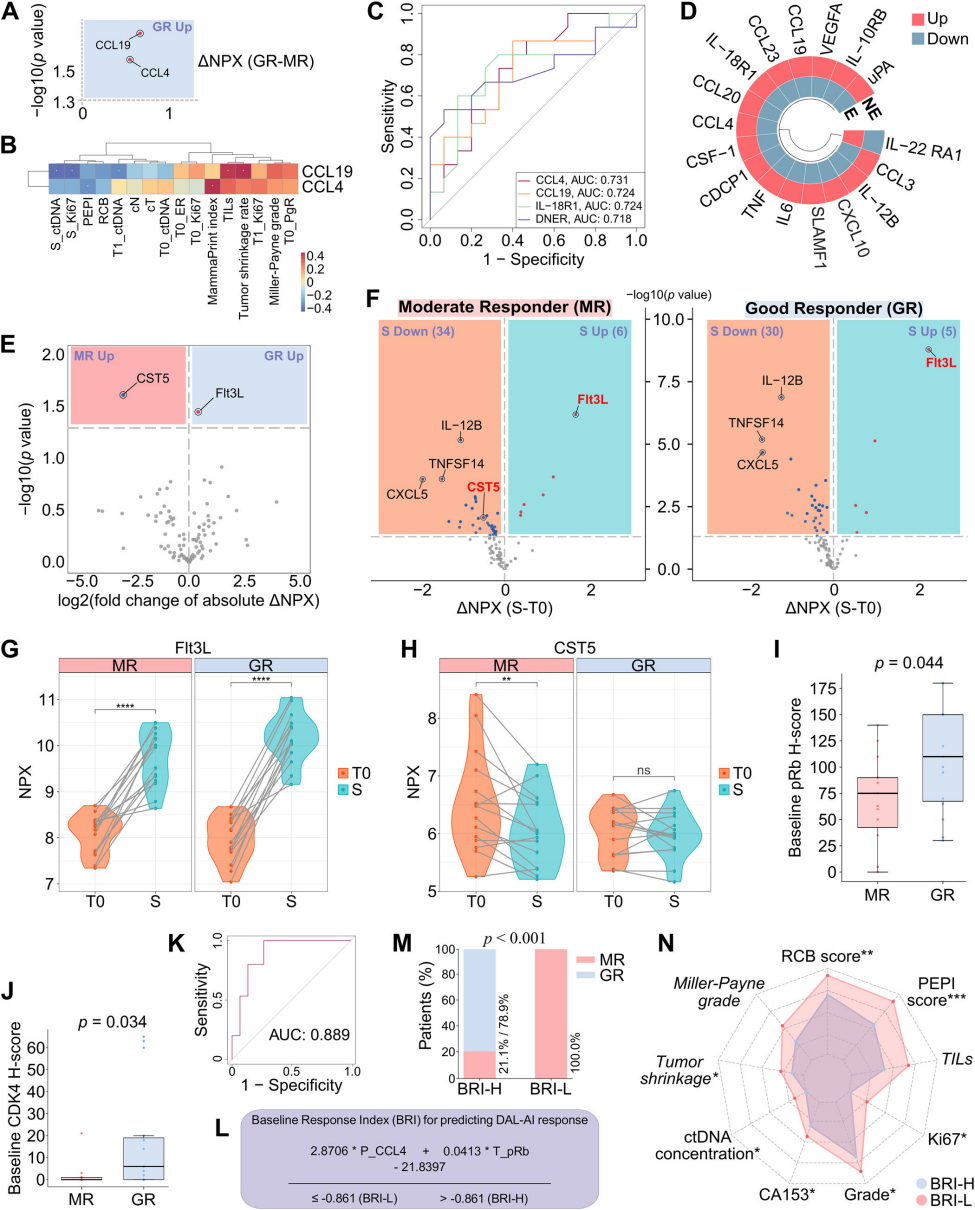
Dynamics of plasma and immunohistochemical protein profiles in good responders (GRs) versus moderate responders (MRs). (A) Simplified volcano plot illustrating significantly different plasma proteins at baseline (T0) between GRs (*n *= 15) and MRs (*n* = 15). (B) Heatmap displaying Spearman correlation analysis between differential proteins and clinicopathological parameters. (C) Receiver operating characteristic (ROC) curve for predicting GR based on baseline plasma proteins, displaying four proteins with *p* values < 0.05. (D) Circular heatmap displaying significantly different plasma proteins at T0 in patients with different ctDNA dynamic trends (elevated [E, *n *= 8] vs. nonelevated [NE, *n *= 22]). (E) Volcano plot comparing absolute level changes in plasma proteins after dalpiciclib therapy between GRs and MRs. (F) Separate volcano plots showing plasma protein level changes after dalpiciclib therapy in MRs (left) and GRs (right). (G and H) Changes in plasma protein levels of Flt3L (G) and CST5 (H) after dalpiciclib therapy in MRs and GRs. (I and J) Baseline *H*‐scores of phosphorylated retinoblastoma protein (pRb) (I) and cyclin‐dependent kinase (CDK) 4 (J) were higher in GRs compared with MRs. (K) ROC curve for predicting GR based on baseline response index (BRI). (L) Formula and cutoff value for BRI. (M) Treatment response in patients stratified by high (*n* = 19) and low (*n* = 11) BRI. (N) Radar plot comparing efficacy and prognostic parameters at surgery between BRI‐high (BRI‐H, *n* = 19) and BRI‐low (BRI‐L, *n* = 11) patients. Miller‐Payne grade, tumor shrinkage rate, and tumor‐infiltrating lymphocyte (TIL) level were italicized to indicate that their raw values were transformed using reciprocal (1/*x*) conversion prior to analysis. All bar values are presented as medians ± interquartile ranges. *p < 0.05, **p < 0.01, ***p < 0.001, ****p < 0.0001. *Abbreviations*: NPX, normalized protein expression; ctDNA, circulating tumor DNA; PEPI, preoperative endocrine therapy prognosis index; RCB, residual cancer burden; ER, estrogen receptor; TIL, tumor‐infiltrating lymphocyte; PgR, progesterone receptor; AUC, area under the curve; CA153, cancer antigen 153.

At T0, the plasma levels of CCL4 and CCL19 proteins were significantly higher in GRs compared with MRs (Figure 6A). These proteins were positively correlated with MammaPrint index, TILs, and tumor shrinkage and negatively correlated with ctDNA concentrations at T1/S, Ki67 expression at S, and PEPI scores (Figure 6B). Based on receiver operating characteristic (ROC) analysis, the levels of four proteins, including CCL4 and CCL19, achieved an area under the curve (AUC) above 0.7 for predicting GR, with all *p* values less than 0.05 (Figure 6C). As aforementioned, patients with CCCA exhibited higher levels of the six differential proteins at T0 compared with patients without CCCA (Figure 3B). We further compared the peripheral immunome at T0 in patients with different ctDNA dynamic trends (i.e., elevated vs. nonelevated). Correspondingly, we identified 17 proteins with significantly different expression levels, among which 16 had higher levels in nonelevated patients, including CCL4 and CCL19 (Figure 6D).

As neoadjuvant therapy progressed, the levels of most proteins exhibited a decreasing trend (Figures 6F and S5). Although no proteins exhibited inverse trajectories (i.e., upregulation in GRs and downregulation in MRs, or vice versa) during neoadjuvant therapy, certain proteins demonstrated significant intergroup differences in the magnitude of their expression changes. For example, increases in plasma levels of Flt3L, a critical growth factor for dendritic cells, were substantially greater in GRs compared with MRs following DAL‐AI treatment (Figure 6E–G). The levels of CST5, which is encoded by the tumor suppressor gene *CST5*, decreased significantly in MRs, whereas they remained virtually unchanged in GRs, resulting in a significant difference in absolute changes in CST5 between the two groups (Figure 6E,H).

#### 2.4.5 Immunohistochemical Biomarkers

The immunohistochemical expression of ER, PgR, phosphorylated Rb (pRb), CDK4, and cyclin D1 proteins was analyzed in tumor tissues. At T0, GRs exhibited significantly higher expression levels of pRb and CDK4 compared with MRs (Figure 6I,J). At both T1 and S, the comparison between GRs and MRs revealed no significant differences in protein expression, albeit a nominally higher expression of PgR at T1 was observed in GRs (*p* = 0.094). Throughout the course of DAL‐AI treatment, PgR expression was significantly decreased in the overall population, whereas CDK4 expression increased significantly. Additionally, pRb and cyclin D1 expression initially rose markedly at T1 but subsequently decreased significantly or nominally at S (Figure S6A). Notably, the extent of these changes did not vary between response status groups, except for CDK4, which significantly increased at T1 only in MRs (Figure S6B).

#### 2.4.6 Baseline Response Index for Predicting DAL‐AI Response

Binary logistic regression was performed based on comprehensive multiomics biomarkers and clinicopathological parameters to develop a baseline response index (BRI) for predicting the therapeutic efficacy of neoadjuvant DAL‐AI. Univariate logistic regression identified candidate predictors, of which two independent proteomic factors were retained in multivariate analysis (*p* < 0.05): plasma CCL4 levels (*β* = 2.8706) and tissue pRb expression (*β* = 0.0413). These parameters were incorporated into the final regression model, which demonstrated excellent discriminative ability for DAL‐AI response, achieving an AUC of 0.889 (95% CI, 0.764–1.000; *p* < 0.001; Figure 6K). The Hosmer–Lemeshow goodness‐of‐fit test confirmed the model's good fit (*p* = 0.817). The optimal cutoff value of −0.861, determined by the maximum Youden index, stratified patients into BRI‐high and BRI‐low groups (Figure 6L). The discriminative performance of BRI was further validated through fivefold cross‐validation and bootstrapping, which yielded AUC values of 0.867 (standard deviation 0.109) and 0.894 (95% CI, 0.755–1.000), respectively.

Higher BRI scores were significantly associated with GR status (Figure 6M). Furthermore, patients with high BRI exhibited significantly lower RCB scores, PEPI scores, ctDNA concentrations, histological tumor grades, Ki67 expression levels, and CA153 levels (Figure 6N), all of which were associated with a more favorable prognosis. BRI‐high patients also showed nominally higher MP grades and TIL levels (*p* < 0.1), and significantly higher tumor shrinkage rates. Further analysis revealed that ctDNA dynamics remained prognostically informative among BRI‐high patients. Within the BRI‐high subgroup (*n* = 19), more patients with ctDNA positivity at T1 had RCB class III compared with those who were ctDNA‐negative at T1, both among patients with ctDNA positivity detected at least once during monitoring (*n* = 11, *p* = 0.015) and in the entire subgroup (*n* = 19, *p* = 0.041) (Figure S7A,B).

## 3 Discussion

To our knowledge, this is the first study to evaluate the feasibility of a ctDNA‐guided approach in the neoadjuvant setting of CDK4/6 inhibitor therapy. This study has demonstrated promising anti‐tumor activity and a manageable safety profile of neoadjuvant dalpiciclib therapy in patients with HR+/HER2− early breast cancer, providing evidence to support the potential integration of CDK4/6 inhibitors into neoadjuvant treatment strategies. More importantly, through serial sample collection and a multiomics profiling strategy, we identified potential biomarkers of DAL‐AI response and subsequently constructed a predictive scoring system. These findings may refine patient selection strategies and enable a more precise identification of responsive subgroups, while also shedding light on potential mechanisms of resistance. Such insights could pave the way for further optimization of CDK4/6 inhibitor‐based neoadjuvant regimens, ultimately improving treatment outcomes in this patient population.

Head‐to‐head studies of neoadjuvant letrozole plus palbociclib/ribociclib versus neoadjuvant chemotherapy in patients with high‐risk HR+/HER2− breast cancer showed lower toxicity without compromising efficacy [10, 11]. However, the results of the CARABELA trial indicated that letrozole together with abemaciclib may be insufficient as neoadjuvant treatment for highly proliferative HR+/HER2− breast cancer, in terms of both pathological and molecular downstaging, as well as sustained Ki67 inhibition [12]. The DARLING‐01 trial, a single‐arm study investigating dalpiciclib combined with letrozole as a neoadjuvant regimen for patients with HR+/HER2− early breast cancer, reported a CCCA rate of 75% (21 out of 28) at 2 weeks and an ORR of 55.2% (16 out of 29) after four cycles [30]. These rates were lower than the 86.7% (26 out of 30) and 62.1% (18 out of 29) observed in the current study for CCCA and ORR, respectively, which may be attributed to the highly selective patient population (luminal B subtype) and the ctDNA‐guided trial design. Furthermore, two patients in our study achieved bpCR, and one patient achieved RCB‐I. With reported pCR rates of 1.4–8.7% for neoadjuvant chemotherapy in HER2‐negative luminal B breast cancer [2], our data suggest that DAL‐AI may elicit a comparable pathological response in patients.

In HR+ breast cancer, Ki67 and its derivative indices, such as CCCA, have shown significant associations with the efficacy and prognosis of neoadjuvant endocrine therapy [16, 17, 18, 19, 20]. Accordingly, Ki67 levels and CCCA are widely used as primary endpoints in clinical trials involving CDK4/6 inhibitors in neoadjuvant settings [4, 13, 20, 31, 32, 33]. Our study aligns with these established practices. Studies have suggested that Ki67 levels after neoadjuvant endocrine therapy correlate with pathological responses, whereas the correlation with radiological responses is less pronounced [34]. Mechanistically, CDK4/6 inhibition halts tumor cell‐cycle progression in the G0–G1 phase, while this cytostatic effect may limit the response rate to CDK4/6 inhibitors [35]. Despite achieving a remarkable CCCA rate, a certain proportion of patients in our study exhibited suboptimal radiological and pathological responses at the end of neoadjuvant therapy. To address this, we further categorized patients based on their CCCA status and imaging response. Notably, GRs exhibited significantly superior outcomes compared with MRs across eight efficacy and prognostic factors. This distinction underscores the importance of a more nuanced response evaluation. In addition, GR/MR stratification yielded well‐balanced group sizes (15 vs. 15) compared with the markedly uneven CCCA‐based grouping (26 vs. 4), thereby enhancing both scientific rigor and statistical reliability for subsequent biomarker discovery and model development in this study.

Accumulating evidence has established a significant correlation between ctDNA positivity and residual tumor burden, both during the early‐to‐mid phase [21, 22, 23] and upon the completion [24] of neoadjuvant therapy. However, this correlation has not been observed in patients with HR+/HER2− breast cancer undergoing neoadjuvant chemotherapy [21]. In contrast, in our investigation of neoadjuvant DAL‐AI therapy, ctDNA was exclusively detectable in MRs but not in GRs at T0^+^, T2, S, and PO. Additionally, among patients with detectable ctDNA, the lack of early‐to‐mid ctDNA clearance could predict MR, RCB III, and PEPI score ≥4. In the future, trials involving ctDNA‐guided neoadjuvant CDK4/6 inhibitor therapy may be further tailored to a highly selective subset of patients with baseline detectable ctDNA, where achieving ctDNA clearance rather than mere decreases at T1 could serve as a more reliable biomarker for guiding subsequent treatment decisions, potentially leading to improved therapeutic outcomes.

Multiple genetic events have been proposed to be linked to sensitivity to CDK4/6 inhibitors [15]. No significant differences in alterations within the cyclin D‐CDK4/6‐Rb pathway were identified between GRs and MRs in our study. However, baseline staining scores for pRb and CDK4 were significantly higher in sensitive patients (i.e., GRs), consistent with previous findings [15, 36]. Beyond cell‐cycle mediators, the copy number variation (CNV) of *GSTM1* emerged as a promising predictive marker, supported by a significant shift in its copy number patterns before and after therapy. Although no prior evidence has directly linked *GSTM1* CNV to CDK4/6 inhibitor response, it is biologically plausible, as the GSTM1 protein is crucial for metabolizing steroid hormones and toxic substances [37], indicating that *GSTM1* amplifications may reduce hormone levels as well as enhance agent metabolism. Additionally, mutations in *CBFB*, *PIK3CA*, *TP53*, and *GATA3* exhibited either group‐specific preferences or persisted posttreatment, potentially correlating with treatment insensitivity. *TP53* mutations conferring resistance to CDK4/6 inhibitors and anti‐estrogens have been suggested, whereas the contribution of mutations in *PIK3CA*, *GATA3*, and *CBFB* to CDK4/6 inhibitor response remains obscure [15].

Using the Olink Target 96 inflammation panel, we found that the peripheral immunome, such as chemokines CCL4 and CCL19, exhibited higher expression levels in patients with favorable treatment outcomes. Additionally, the plasma levels of Flt3L, a critical growth factor for dendritic cells that activates endogenous T cells and enhances anti‐tumor responses [38], were increased substantially more in GRs compared with MRs following DAL‐AI treatment. Similarly, at the genomic level, activating events involving innate immune response pathway genes were more frequently observed in GRs. Therefore, patients with robust anti‐tumor immune responses may derive greater benefit from CDK4/6 inhibitor therapy. In turn, preclinical studies have shown that CDK4/6 inhibitors can enhance tumor antigen presentation, activate T cells, augment cytotoxic T‐cell‐mediated killing, and improve the efficacy of anti‐PD‐1 immunotherapy [39, 40, 41]. These findings suggest a potential synergistic effect and a promising therapeutic combination of CDK4/6 inhibition and immunotherapy.

We developed a predictive scoring system, the BRI, a composite score based on plasma CCL4 levels and tumor tissue pRb expression to better integrate candidate biomarkers from segregation analyses with putative predictive effects. Despite the limited sample size, the model demonstrated robust predictive power for treatment efficacy, achieving an AUC of 0.889. The BRI‐high subgroup, defined by the established cutoff, showed significantly better treatment response and improved prognostic potential. Notably, early ctDNA dynamics further refined risk stratification within the BRI‐high subgroup. Those who were ctDNA‐negative at T1 derived greater benefit from the four‐cycle DAL‐AI regimen, whereas ctDNA‐positive and BRI‐low patients may be candidates for alternative therapies. This two‐step biomarker‐guided strategy, combining BRI‐based baseline stratification with ctDNA monitoring during treatment, may support more precise therapeutic adaptation and merits prospective validation.

## 4 Limitations of the Study

The current study possesses several limitations. First, the single‐arm trial design and modest sample size may introduce biases and limit statistical support for our findings, thereby restricting the applicability to broader populations. Second, the lack of transcriptome data hinders the comprehensive interpretation of genomic discoveries and the integration of multiomics datasets. Thirdly, our focus on resistance in patients treated with CDK4/6 inhibitor plus AI may obscure the mechanisms specific to CDK4/6 inhibitors. For instance, *PIK3CA* mutations, potentially predictive of DAL‐AI resistance, may primarily affect endocrine therapy rather than CDK4/6 inhibitors [42]. Finally, although the GR/MR classification in this study was significantly associated with multiple prognostic indicators (including RCB, PEPI, Ki67, and others), its prognostic relevance in real‐world settings remains to be validated. Only with such validation can the identified biomarkers and the constructed predictive model gain practical clinical significance.

## 5 Conclusion

In conclusion, our study evaluated the potential of ctDNA‐directed neoadjuvant CDK4/6 inhibitor therapy for patients with operable HER2‐negative luminal B breast cancer and demonstrated the biological and clinical activity of dalpiciclib plus AI with manageable toxicity. The candidate biomarkers included early ctDNA clearance, *GSTM1* status, MammaPrint index, plasma CCL4/CCL19 levels, and tissue pRb/CDK4 staining. We developed a predictive model based on plasma CCL4 levels and tissue pRb staining, with high efficacy in predicting treatment response, and stratified patients by the likelihood of clinical benefit. Notably, incorporating early ctDNA dynamics enabled refined risk stratification, facilitating adaptive treatment strategies by distinguishing likely responders from those who might benefit from alternative therapies. Our findings strengthen the evidence supporting CDK4/6 inhibitors in the neoadjuvant setting and highlight the value of biomarker‐guided strategies for optimizing treatment.

## 6 Methods

### 6.1 Trial Design

DANCER is a prospective, single‐center, single‐arm, ctDNA‐directed, phase II trial. Eligible postmenopausal patients were administered neoadjuvant AI (either anastrozole 1 mg daily or letrozole 2.5 mg daily) in combination with dalpiciclib (150 mg daily for 3 weeks on, 1 week off). Premenopausal patients received AI therapy 14 days prior to the first dose of dalpiciclib (Cycle 0), concurrent with a GnRH agonist administered every 28 days from the first day of Cycle 0.

Two weeks after DAL‐AI initiation, the patients underwent a second core needle biopsy and peripheral venous blood sampling to assess Ki67 and ctDNA levels at this time point (T1). Subsequently, patients achieving CCCA or decreases in ctDNA levels compared with baseline proceeded to continued DAL‐AI treatment in repeated cycles, totaling 15 weeks (four cycles) before surgery; dalpiciclib doses could be adjusted downward based on patient toxicity. Conversely, patients without CCCA and with elevated ctDNA levels were assigned to either an alternative neoadjuvant treatment regimen or surgical intervention (Figure 1A).

A breast tissue marker clip was placed during the second core needle biopsy at T1. Ultrasound was conducted at baseline (T0), the end of Cycle 0 before the first dalpiciclib dose (T0^+^), T1, and the last day of each cycle. MRI assessments were conducted at T0, mid‐therapy (8 weeks after DAL‐AI administration, T2), and immediately before surgery (S).

Surgery was scheduled 1 day after the last dalpiciclib dose to minimize the risk of Ki67 rebound. Postoperative adjuvant treatment strategies were determined according to RCB, treatment response, MammaPrint index, and local practice guidelines. In cases of unsatisfactory treatment responses or severe toxicity, the investigator decided whether to discontinue treatment and proceed immediately to surgery. Patients had the right to withdraw from the study at any time for any reason.

### 6.2 Patients and Sample Collection

Patients eligible for the DANCER trial were aged between 18 and 75 years, with histologically confirmed, previously untreated, unilateral luminal B invasive breast cancer (ER ≥ 10%, HER2‐negative, with Ki67 ≥ 20% and/or PgR < 20%). Patients were required to have operable stage I–III breast cancer with a diameter of ≥2 cm as assessed by MRI, and a baseline Eastern Cooperative Oncology Group performance status of 0–1. The exclusion criteria encompassed inflammatory breast cancer, stage IV disease, occult or bilateral breast cancer, and pregnancy. Individuals diagnosed with any other malignancy within the preceding 5 years were also deemed ineligible. Patients exhibiting inadequate hemopoietic, cardiac, hepatic, or renal function were also excluded from participation. The study adhered to the principles of Good Clinical Practice and the Declaration of Helsinki. Written informed consent was obtained from all participants prior to enrollment. The study protocol was approved by the Institutional Review Board of the Second Affiliated Hospital of Zhejiang University School of Medicine (approval number: 2022‐0500). The study was registered with ClinicalTrials.gov (NCT05640778).

Serial trial‐specific plasma samples were collected at T0, T0^+^, T1, T2, S, and 2–4 weeks PO for ctDNA analyses, with samples at T0, T1, and S also subjected to proteomic profiling (Figure 1B). Tumor tissues were collected at T0, T1, and S to evaluate of the MammaPrint index, somatic variation profiling, and perform histopathological and immunohistochemical analyses. Exploratory analyses of comprehensive biomarkers were performed.

### 6.3 Efficacy and Safety Evaluation

The primary endpoint was the CCCA rate at T1. CCCA was defined as a Ki67 expression level ≤ 2.7%. Immunohistochemical staining for Ki67 was performed on formalin‐fixed and paraffin‐embedded (FFPE) breast tumor samples at the participating hospital pathology laboratory using an anti‐Ki67 antibody (clone MIB1; 1:300; ZSGB, Beijing, China). The samples were derived from either core needle biopsies or surgical specimens and were blindly evaluated by two pathologists according to standard scoring guidelines [43].

The secondary endpoints included clinical response, pathological response, ctDNA clearance, safety, 5‐year disease‐free survival, and 5‐year overall survival. Clinical response (by MRI and by palpation) was assessed as per the Response Evaluation Criteria in Solid Tumors version 1.1. Pathological response was determined using the MP [44] and RCB grading systems [45], as well as PEPI scores [46]. bpCR was defined as the complete eradication of invasive cancer cells in the breast after neoadjuvant therapy (ypT0/is, i.e., MP grade of 5), while tpCR (i.e., RCB‐0) further required the absence of axillary lymph node metastases (ypN0) in addition to bpCR. Tumors were located by researchers and pathologists based on baseline skin markings, breast tissue marker clips, and intraoperative mammography. Standardized tissue sampling was performed on surgically excised specimens following the sampling principles of the RCB assessment system [47]. Imageological evaluations were independently conducted by two radiologists, and pathological evaluations by two pathologists, with discordant cases being reviewed by a third specialist. ctDNA clearance was established as the state of negativity in patients who were initially ctDNA‐positive. Safety profiles were assessed (through laboratory assessments, electrocardiogram, performance status, subject diary card, and vital signs) on Days 1, 7, 15, and 28 of Cycle 1, and monthly thereafter, according to the Common Terminology Criteria for Adverse Events version 5.0. Survival outcomes will be addressed in the future once mature data become available for analysis.

### 6.4 Immunohistochemistry

In addition to Ki67 staining, immunohistochemical staining was conducted for ER, PgR, HER2, CDK4, pRb, and cyclin D1. Sections measuring 4 µm were obtained from FFPE tissue and stained with both hematoxylin and eosin, as well as immunohistochemical stains. The expression of ER, PgR, and HER2 was determined locally in accordance with ASCO/CAP guidelines. *H*‐scores were calculated for ER, PgR, CDK4, pRb, and cyclin D1 using the following formula: *H*‐score = (% of cells with weak staining [1+]) × 1 + (% with moderate staining [2+]) × 2 + (% with strong staining [3+]) × 3, yielding a total score ranging from 0 to 300. The antibodies used were ER (Clone SP1; ready to use (RTU); Ventana‐Roche Diagnostics, Tucson, USA), PgR (Clone 1E2; RTU; Ventana‐Roche Diagnostics), HER2 (Clone 4B5, RTU; Ventana‐Roche Diagnostics), CDK4 (Clone D9G3E; 1:400; Cell Signaling Technology, Danvers, USA), pRb (Clone D20B12; 1:200; Cell Signaling Technology), and cyclin D1 (Clone SP4; 1:100; Abcam, Cambridge, USA).

### 6.5 DNA Extraction, Sequencing, and Data Processing

Genomic DNA (gDNA) of the tumor was obtained from FFPE tumor specimens with tumor cellularity exceeding 20% using the MagPure FFPE DNA Kit (Magen, China). Cell‐free DNA (cfDNA) was extracted from plasma samples using the MagMAX Cell‐Free DNA Isolation Kit (Thermo Fisher Scientific, USA). Meanwhile, the gDNA of the white blood cells, isolated using the TGuide S32 Magnetic Blood Genomic DNA Kit (TIANGEN, China), was simultaneously prepared for the analysis of germline mutations and clonal hematopoiesis filtration. DNA concentration was measured using Qubit dsDNA HS (High Sensitivity) Assay Kit (Thermo Fisher Scientific), with DNA quality evaluated by Agilent 2100 Bioanalyzer (Agilent, USA). cfDNA libraries were prepared by KAPA HyperPrep PCR‐free Kit (KAPA Biosystems, USA), while gDNA libraries were prepared by Watchmaker DNA Library Prep Kit (Watchmaker Genomics, USA). DNA libraries from tissue samples and plasma samples were captured with a custom‐designed panel that covers 769 cancer‐related genes (Table S6). The captured library was sequenced on the MGI DNBSEQ‐T7 platform, generating 2×100 bp paired‐end reads.

After removing low‐quality reads by Trimmomatic (v0.36) [48], clean reads were aligned to the human reference genome (Hg19, NCBI Build 37.5) with Burrows‐Wheeler Aligner (v0.7.17) [49]. Other bioinformatics tools employed for processing next‐generation sequencing (NGS) data included the Picard toolkit (v2.23.0) [50] for marking duplicates, the Genome Analysis Toolkit (v3.7) [51] for realignment, VarDict (v1.5.1) [52] and FreeBayes (v1.2.0) [53] for calling SNVs and compound heterozygous mutations, and Lumpy [54] for structural variant detection. CNVs were paired called via software ctCNV [55] with a copy number threshold of 4 for CNV gain and 1 for CNV loss. Sentieon software (genomics‐201911) was also utilized to improve the detection rate of plasma mutations. The variants were annotated through ANNOVAR [56].

### 6.6 Somatic Mutation Filtering and ctDNA Analysis

Somatic mutations in tumor tissues were obtained after filtering germline mutations and the final somatic mutations used for the following analysis were selected based on the following criteria: (i) known hotspot mutations with a frequency of ≥1% and other mutations with a frequency of ≥2%; (ii) not located in intergenic regions or intronic regions and not synonymous SNVs; (iii) support reads ≥5; and (iv) allele frequency ≤0.2% in the Exome Aggregation Consortium [57] and Genome Aggregation Database [58].

Somatic mutation analysis of plasma followed a tumor‐informed strategy. Germline and hematopoietic‐origin mutations were excluded using a paired normal sample, and only tumor‐specific somatic mutations were retained for downstream analysis. These mutations were then used to track changes in subsequent plasma samples. Filtering criteria were applied to obtain reliable mutations in tumor samples: (i) the sequencing depth was more than 100; (ii) the variant allele frequency (VAF) threshold of SNV was 4% and that of InDels was 5%. A statistical test was performed on each candidate ctDNA mutation against an internal reference background library to eliminate residual artifacts. The background library consisted of over 1000 plasma samples with matched tissue and peripheral blood cell samples from patients of various stages and cancer types. Only mutations with *p*‐values of less than 0.01 were considered reliable. Combined *p* values were further introduced to define a positive plasma sample with a cutoff *p *< 0.01. The combined *p* value was calculated using the following formula: $\;P_{\mathrm{sample}}=C_{m}^{k}\Pi P_{i}$, where *m* of the combination coefficient (*C*) represents the number of variants tracked and *k* represents the number of variants that passed a variant level threshold.

ctDNA concentration was quantified in terms of haploid genome equivalent (hGE) per milliliter of plasma (hGE/mL), and calculated as follows [59]: $\frac{\mathrm{mean}\;\mathrm{VAF}\times \mathrm{cfDNA}\;\mathrm{concentration}\;(\mathrm{pg}/\mathrm{mL})\;}{3.3}$. In this formula, cfDNA concentration (pg/mL) was derived by dividing the total extracted cfDNA yield (pg) by the plasma volume used for extraction (mL). Mean VAF (%) was calculated as the average VAF measured in all quality control‐passing targets.

### 6.7 Multigene Assays

The 70‐gene signature (MammaPrint) and the 80‐gene molecular subtyping signature (BluePrint) were analyzed in a blinded manner at Genecast Laboratory, a Reference Laboratory Partner certified by Agendia, without integration of patients’ clinical characteristics. The multigene expression profile was assessed in the FFPE breast samples collected at baseline via core needle biopsy (*n* = 30) or during surgery through surgical resection (*n* =  4). FFPE RNA was extracted using the Qiagen RNeasy FFPE Kit (Qiagen, Germany). The concentration of RNA was measured by Nanoready Touch (Suizhen, China), while the quality of RNA was assessed by Agilent 4200 BioAnalyzer (Agilent). RNA was used to construct a library employing the MammaPrint and BluePrint Breast Cancer Recurrence and Molecular Subtyping Kit (Agendia, Netherlands). The target enrichment workflow utilized ultra‐long 120‐mer biotinylated cRNA baits to capture MammaPrint and BluePrint genes, enriching them from an NGS genomic fragment library. The captured library was sequenced on the Illumina MiseqDX instrument as per the manufacturer's protocols, producing single‐end reads with 150 bp length. Tumors were classified as high or low genomic risk.

BluePrint stratifies tumors into three intrinsic molecular subtypes: luminal‐type, HER2‐type, and basal‐type. Within the luminal subgroup, MammaPrint further classifies tumors into luminal A (MammaPrint low risk) and luminal B (MammaPrint high risk) [27].

### 6.8 Plasma Immune Proteomics

Plasma immune proteomic analysis was performed blinded to clinicopathological data at the LC‐Bio Technology Co., Ltd. laboratory in Hangzhou, China. The analysis utilized the Olink Target 96 inflammation panel (Olink Proteomics, Sweden) to measure the levels of 92 marker proteins involved in key immune and inflammatory pathways (listed in Table S7) using proximity extension assay technology [60]. Briefly, pairs of oligonucleotide‐labeled antibody probes were bound specifically to their target proteins, generating double‐stranded DNA amplicons upon successful binding by paired antibodies. These amplicons served as indicators of protein levels and could be quantified accordingly. Quality control and normalization of the data were performed using an internal extension control and an interplate control to adjust for intra‐ and inter‐run variations. The final read‐out protein levels were presented in normalized protein expression (NPX) values, which were arbitrary units on a log2 scale. A higher NPX value corresponded to a higher protein expression level, with an increase of 1 unit representing a doubling of the protein concentration.

### 6.9 Biomarker Analysis and Logistic Regression

In terms of biomarkers, patients who achieved CCCA at both T1 and S, along with a concurrent objective response on MRI at S, were classified as GRs (*n* = 15). The remaining patients were categorized as MRs (*n* = 15). Subsequently, we compared the molecular landscapes and dynamic changes between the two groups to identify candidate biomarkers for DAL‐AI response. All 205 multiomics biomarkers and clinicopathological parameters were included in the univariate logistic analysis, and those with a *p* value of less than 0.05 were subsequently incorporated into the multivariate binary logistic regression analysis. No more than two factors were allowed in the multivariate analysis due to sample size limitations. Through pairwise combinations, multivariate regression equations were constructed, and only equations in which both factors and the constant term exhibited *p* values of less than 0.05 were retained. The corresponding factors were used to construct a predictive scoring model to estimate the probability of GR. The model was developed by incorporating the regression coefficients (*β*) of each independent predictor and the constant identified in the multivariate analysis. The cutoff value was determined based on the maximum Youden index derived from the ROC curve. The discriminative ability of the risk score was validated using fivefold cross‐validation and bootstrapping.

### 6.10 Statistical Analysis

All patients who underwent a second core needle biopsy at T1 were included in the analysis of efficacy, safety, and biomarkers. The Shapiro–Wilk test and Kolmogorov–Smirnov test were utilized to assess the distribution types of the data. The 95% CIs for the rates of CCCA, along with various other efficacy indices, were calculated using the Clopper‐Pearson method. For comparisons between two groups, Student's *t*‐tests were used for normally distributed continuous variables and the Wilcoxon rank‐sum test for non‐normally distributed ones. For paired data across different time points, paired *t*‐tests or paired Wilcoxon signed‐rank tests were applied. Categorical variable relationships were evaluated using Fisher's exact test, and correlated proportions were analyzed with McNemar test or McNemar–Bowker test. Spearman's rank correlation analysis was used to calculate correlation coefficients. The OR for gene set alteration analysis between GRs and MRs was calculated as follows: $\mathrm{OR}=\frac{\left(\frac{n\;\mathrm{of}\;\mathrm{GRs}\;\mathrm{with}\;\mathrm{gene}\;\mathrm{set}\;\mathrm{alteration}}{n\;\mathrm{of}\;\mathrm{GRs}\;\mathrm{without}\;\mathrm{gene}\;\mathrm{set}\;\mathrm{alteration}}\right)}{\left(\frac{n\;\mathrm{of}\;\mathrm{MRs}\;\mathrm{with}\;\mathrm{gene}\;\mathrm{set}\;\mathrm{alteration}}{n\;\mathrm{of}\;\mathrm{MRs}\;\mathrm{without}\;\mathrm{gene}\;\mathrm{set}\;\mathrm{alteration}}\right)}$. When calculating ORs with zero counts, a continuity correction of +0.5 was applied to all cell counts. ROC curves and AUC values were plotted and calculated using the roc_curve function from the scikit‐learn library. Statistical analyses were performed using Python software (version 3.9.15), R software (version 4.3.1), and SPSS software (version 26.0). A two‐sided *p* value < 0.05 was considered statistically significant.

## Author Contributions

Y.D.C., L.Q.P., Y.X.Z., and F.B.H. participated in the design of the trial. Y.D.C., Y.X.Z., H.H.C., C.P., Y.H., Y.S., and S.J.W. recruited and treated patients. F.B.H., S.Q.T., W.Q., H.H., H.W., and M.D.M. were responsible for tumor evaluations. Y.X.Z., L.S., S.J.W., and X.N.G. collected data. Y.X.Z. and Z.Y.Z. contributed to data analysis and interpretation. H.H.C. and Y.X.Z. drafted the manuscript. Z.W., J.Z., J.F., W.K.M., and T.M. generated the original figures. L.Q.P., Y.X.Z., Z.Y.Z., and J.Z. critically revised the text and figures. Y.D.C. and L.Q.P. supervised the research and led discussions. All authors have read and approved the final manuscript.

## Ethics Statement

This study was reviewed and approved by the Institutional Review Board of the Second Affiliated Hospital of Zhejiang University School of Medicine (approval number: 2022‐0500). The study was registered with ClinicalTrials.gov (NCT05640778). Written informed consent was obtained from all participants prior to enrollment. This study complied with the Declaration of Helsinki. The patient data were maintained with confidentiality.

## Conflicts of Interest

Authors Z.W., J.Z., and T.M. are employees of Genecast Biotechnology Co., Ltd., and J.F. and W.K.M. are employees of LC‐Bio Technology Co., Ltd., but none of them have any relevant financial or nonfinancial interests to disclose. The other authors declare no conflicts of interest.

## Supporting information



Figure S1. Tumor shrinkage rate, breast cancer‐specific survival (BCSS) preoperative endocrine therapy prognosis index (PEPI) scores, and residual cancer burden (RCB) scores in patients with complete cell cycle arrest (CCCA, *n *= 26) and without CCCA (*n *= 4) at 2 weeks (T1) after the initiation of dalpiciclib treatment. Related to Figure 2.
Figure S2. Circulating tumor DNA (ctDNA) clearance and ctDNA status are associated with preoperative endocrine therapy prognosis index (PEPI) and residual cancer burden (RCB) scores. Related to Figure 4. (A and B) Association of ctDNA clearance with PEPI (A) and RCB (B). Clear ≤ T1: ctDNA clearance at T0+ or T1, without rebound; clear ≤ T2: ctDNA clearance at T0+, T1, or T2, without rebound; clear ≤ S: ctDNA clearance at T0+, T1, T2, or S, without rebound. (C and D) PEPI score (C) and RCB score (D) in patients grouped according to ctDNA status at different time points: T0 (17/13), T0+ (2/12), T1 (9/21), T2 (3/14), S (3/27), and PO (2/28), with values representing ctDNA‐positive/negative patient counts, respectively. *Abbreviations*: OR: odds ratio; RFS: relapse‐free survival; BCSS: breast cancer‐specific survival; T0: baseline; T0+: the end of Cycle 0 before the first dalpiciclib dose, only for premenopausal patients; T1: 2 weeks after dalpiciclib treatment initiation; T2: 8 weeks after dalpiciclib treatment initiation; S: surgery; PO: 2–4 weeks postoperatively.
Figure S3. Dynamics of genetic variations after neoadjuvant therapy. Related to Figure 5. (A) Significant decreases in variation counts in the overall population (*n *= 30) after neoadjuvant therapy. (B) Wild‐type *CBFB* was significantly correlated with sustained complete cell cycle arrest (CCCA) and concurrent circulating tumor DNA (ctDNA) clearance after neoadjuvant therapy. (C) Sankey diagram illustrating dynamic changes in *GSTM1* copy numbers after neoadjuvant therapy. The copy number of *GSTM1* was categorized into deletion (loss), normal, and amplification (gain). (D) Pattern of the top 11 most altered variant types after neoadjuvant therapy, grouped by treatment response (good responder [GR], *n *= 15; moderate responder [MR], *n *= 15). T0: baseline; S: surgery; mut: mutation; wt: wild type.
Figure S4. Longitudinal dynamics of pathway alterations during neoadjuvant therapy. Related to Figure 5. (A) Analysis of alterations in gene sets derived from the Gene Ontology Biological Process ontology between Good Responders and Moderate Responders at 2 weeks postdalpiciclib treatment (T1). (B) Analysis of alterations in gene sets derived from the Gene Ontology Biological Process ontology between Good Responders and Moderate Responders at surgery (S). OR: odds ratio.
Figure S5. Dynamics of plasma proteins in 28 patients with matched samples at all three time points, showing significant changes during neoadjuvant therapy. Related to Figure 6.
Figure S6. Dynamics of immunohistochemical biomarkers after neoadjuvant therapy. Related to Figure 6. (A) H‐scores of five immunohistochemical biomarkers across the time points in the overall population (*n *= 30). (B) Expression levels of CDK4 at baseline (T0) and 2 weeks postdalpiciclib treatment (T1) in patients with different treatment responses (good responder [GR], *n *= 15; moderate responder [MR], *n *= 15). *Abbreviations*: S: surgery; ER: estrogen receptor; PgR: progesterone receptor; pRb: phosphorylated retinoblastoma protein; CDK: cyclin‐dependent kinase.
Figure S7. Prognostic value of circulating tumor DNA (ctDNA) positivity at 2 weeks postdalpiciclib treatment (T1) in the baseline response index (BRI)‐high subgroup. Related to Figure 6. (A) Comparison of residual cancer burden (RCB)‐III proportions between patients with ctDNA clearance (T1‐negative) and persistence (T1‐positive) at T1 among BRI‐high patients with ctDNA positivity detected at least once during monitoring (*n *= 11). (B) RCB‐III distribution by ctDNA status at T1 in the entire BRI‐high subgroup (*n *= 19). *p* Values were determined using Fisher's exact test.
Table S1. Summary of common treatment‐emergent adverse events (TEAEs).
Table S2. Association between the timing of neutropenia occurrence and dalpiciclib dose reduction.
Table S3. Baseline patient characteristics according to response status.
Table S4. Molecular Signatures Database (MSigDB) Gene Ontology gene sets related to response status (*p *< 0.1).
Table S5. Activating events involving genes annotated to the activation of innate immune response gene set observed in Good Responders at baseline.
Table S6. The list of 769 genes included in the NGS panel.
Table S7. The list of 92 proteins included in Olink Target 96 inflammation panel.

## Data Availability

The datasets generated and/or analyzed during the current study are available in GSA for Human at https://ngdc.cncb.ac.cn/gsa‐human/, reference number HRA009865.
