# Supplementary material for: Efficacy, Safety, and Biomarkers of Neoadjuvant Dalpiciclib (CDK4/6 inhibitor) plus Aromatase Inhibitors in Operable HER2‐Negative Luminal B Breast Cancer: A Prospective, Single‐Center, Single‐Arm, Phase II Trial (DANCER)
Source: MedComm (2020). 2025 Oct 11;6(10):e70402. doi: 10.1002/mco2.70402 (PMC12514558; doi:10.1002/mco2.70402)
Supplement: Supplementary file 1 — Figure S1. Tumor shrinkage rate, breast cancer‐specific survival (BCSS) preoperative endocrine therapy prognosis index (PEPI) scores, and residual cancer burden (RCB) scores in patients with complete cell cycle arrest (CCCA, n = 26) and without CCCA (n = 4) at 2 weeks (T1) after the initiation of dalpiciclib treatment. Related to Figure 2. Figure S2. Circulating tumor DNA (ctDNA) clearance and ctDNA status are associated with preoperative endocrine therapy prognosis index (PEPI) and residual cancer burden (RCB) scores. Related to Figure 4. (A and B) Association of ctDNA clearance with PEPI (A) and RCB (B). Clear ≤ T1: ctDNA clearance at T0+ or T1, without rebound; clear ≤ T2: ctDNA clearance at T0+, T1, or T2, without rebound; clear ≤ S: ctDNA clearance at T0+, T1, T2, or S, without rebound. (C and D) PEPI score (C) and RCB score (D) in patients grouped according to ctDNA status at different time points: T0 (17/13), T0+ (2/12), T1 (9/21), T2 (3/14), S (3/27), and PO (2/28), with values representing ctDNA‐positive/negative patient counts, respectively. Abbreviations: OR: odds ratio; RFS: relapse‐free survival; BCSS: breast cancer‐specific survival; T0: baseline; T0+: the end of Cycle 0 before the first dalpiciclib dose, only for premenopausal patients; T1: 2 weeks after dalpiciclib treatment initiation; T2: 8 weeks after dalpiciclib treatment initiation; S: surgery; PO: 2–4 weeks postoperatively. Figure S3. Dynamics of genetic variations after neoadjuvant therapy. Related to Figure 5. (A) Significant decreases in variation counts in the overall population (n = 30) after neoadjuvant therapy. (B) Wild‐type CBFB was significantly correlated with sustained complete cell cycle arrest (CCCA) and concurrent circulating tumor DNA (ctDNA) clearance after neoadjuvant therapy. (C) Sankey diagram illustrating dynamic changes in GSTM1 copy numbers after neoadjuvant therapy. The copy number of GSTM1 was categorized into deletion (loss), normal, and amplification (gain). (D) Patter [file MCO2-6-e70402-s001.pdf]

Supplementary information

Efficacy, safety, and biomarkers of neoadjuvant dalpiciclib (CDK4/6 inhibitor) plus aromatase inhibitors in operable HER2-negative luminal B breast cancer: a prospective, single-center, single-arm, phase II trial (DANCER)

Yunxiang Zhou[1,2,#], Zhiyun Zhang[1,2,#], Huihui Chen[1,#], Fengbo Huang[3], Lu Shen[1,2], Siqi Tao[3], Wei Qian[4], Hui Hong[4], Chi Pan[1], Ze Wang[5], Jiao Zhang[5], Yue Hu[1], Yong Shen[1], Jun Fu[6], Weikang Mao[6], Shijie Wu[1,2], Xianan Guo[1,2], Hui Wang[7], Mindi Ma[8], Ting Ma[5], Liqiang Pan[9,10,*], Yiding Chen[1,2,*]

1 Department of Breast Surgery and Oncology, The Second Affiliated Hospital, Zhejiang University School of Medicine, Hangzhou, Zhejiang, China

2 Cancer Institute (Key Laboratory of Cancer Prevention and Intervention, China National Ministry of Education), The Second Affiliated Hospital, Zhejiang University School of Medicine, Hangzhou, China

3 Department of Pathology, The Second Affiliated Hospital, Zhejiang University School of Medicine, Hangzhou, Zhejiang, China

4 Department of Radiology, The Second Affiliated Hospital, Zhejiang University School of Medicine, Hangzhou, Zhejiang, China

5 Genecast Biotechnology Co., Ltd, Wuxi, Jiangsu, China

6 LC-Bio Technology Co., Ltd, Hangzhou, China

7 Department of Pathology, Zhejiang Cancer Hospital, Hangzhou, Zhejiang, China

8 Department of Radiology, The First Affiliated Hospital of Zhejiang Chinese Medical University (Zhejiang Provincial Hospital of Chinese Medicine), Hangzhou, Zhejiang, China

9 Laboratory of Precision Medicine and Biopharmaceuticals, College of Pharmaceutical Sciences, Zhejiang University, Hangzhou, Zhejiang, China

10 Department of Pharmacy, The Second Affiliated Hospital, Zhejiang University School of Medicine, Hangzhou, Zhejiang, China

Yunxiang Zhou, Zhiyun Zhang and Huihui Chen contributed equally to this manuscript.

***Correspondence**

Yiding Chen, Email: ydchen@zju.edu.cn.

Liqiang Pan, Email: panliqiang@zju.edu.cn.

Supplementary Figures

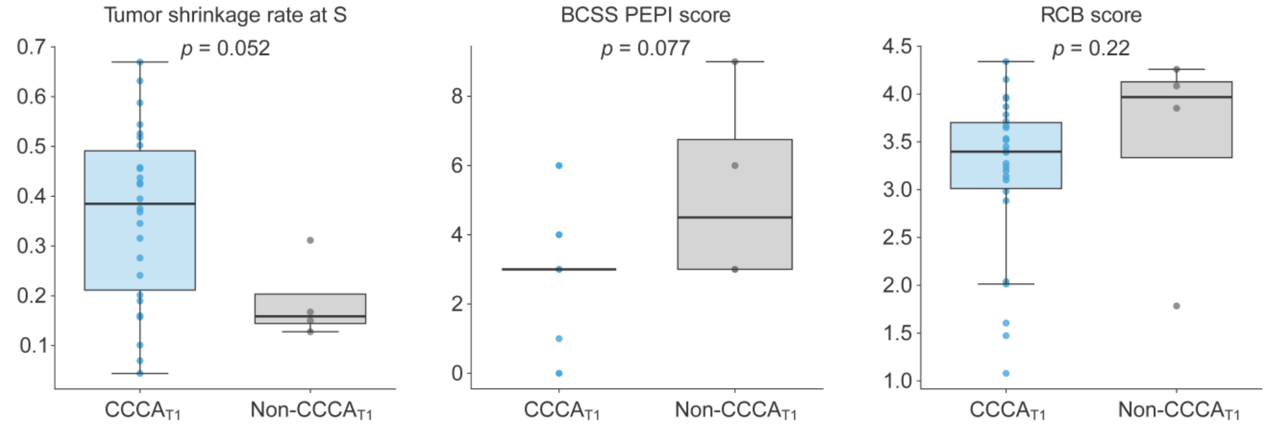

Figure S1. Tumor shrinkage rate, breast cancer-specific survival (BCSS) preoperative endocrine therapy prognosis index (PEPI) scores, and residual cancer burden (RCB) scores in patients with complete cell cycle arrest (CCCA, *n* = 26) and without CCCA (*n* = 4) at 2 weeks (T1) after the initiation of dalpiciclib treatment. Related to Figure 2.

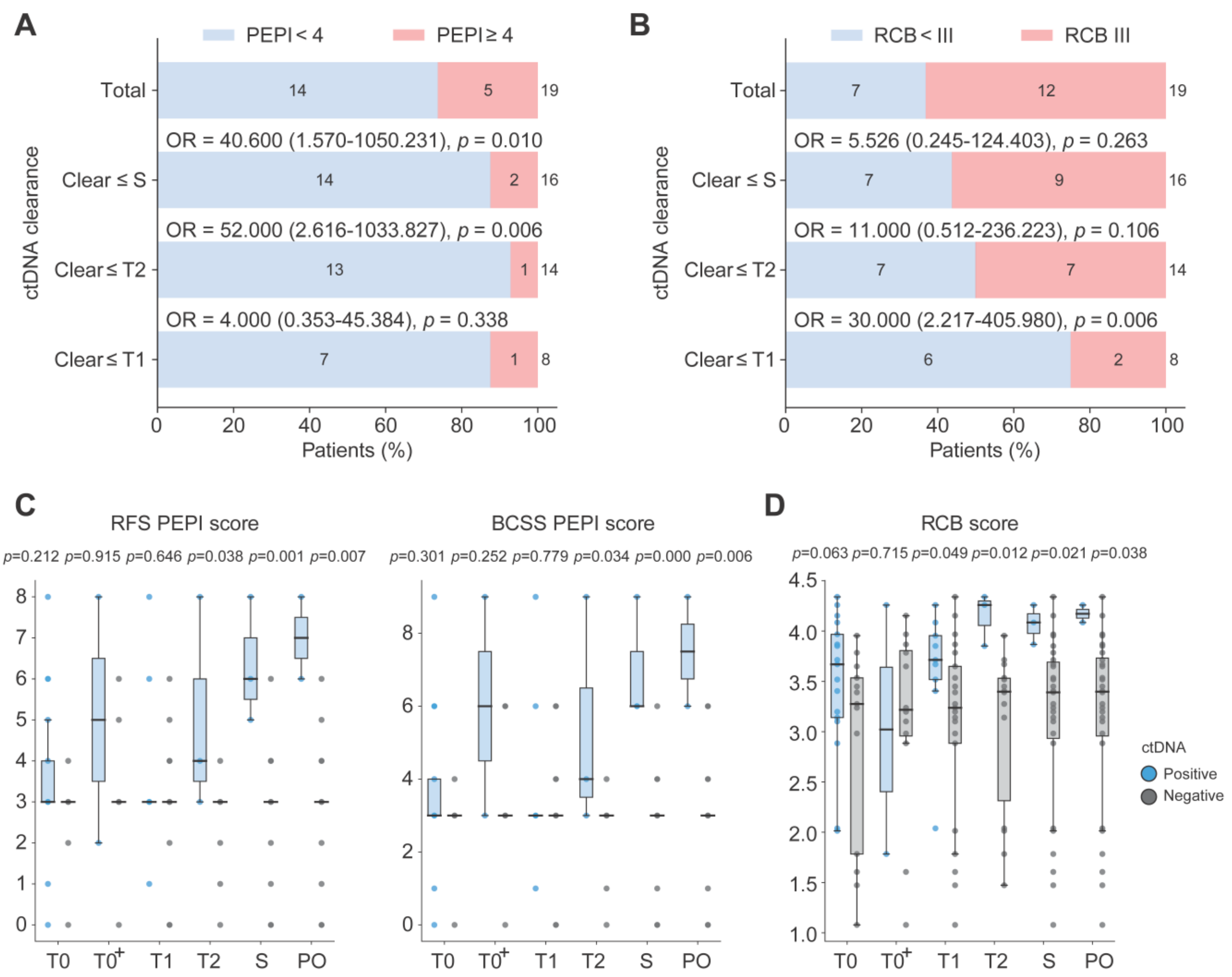

Figure S2. Circulating tumor DNA (ctDNA) clearance and ctDNA status are associated with preoperative endocrine therapy prognosis index (PEPI) and residual cancer burden (RCB) scores. Related to Figure 4.

(A–B) Association of ctDNA clearance with PEPI (A) and RCB (B). Clear ≤ T1: ctDNA clearance at $T0^+$ or T1, without rebound; clear ≤ T2: ctDNA clearance at $T0^+$, T1, or T2, without rebound; clear ≤ S: ctDNA clearance at $T0^+$, T1, T2, or S, without rebound.

(C–D) PEPI score (C) and RCB score (D) in patients grouped according to ctDNA status at different time points: T0 (17/13), $T0^+$ (2/12), T1 (9/21), T2 (3/14), S (3/27), and PO (2/28), with values representing ctDNA-positive/negative patient counts, respectively.

OR: odds ratio; RFS: relapse-free survival; BCSS: breast cancer-specific survival; T0: baseline; $T0^+$: the end of Cycle 0 before the first dalpiciclib dose, only for premenopausal patients; T1: 2 weeks after dalpiciclib treatment initiation; T2: 8 weeks after dalpiciclib treatment initiation; S: surgery; PO: 2–4 weeks postoperatively.

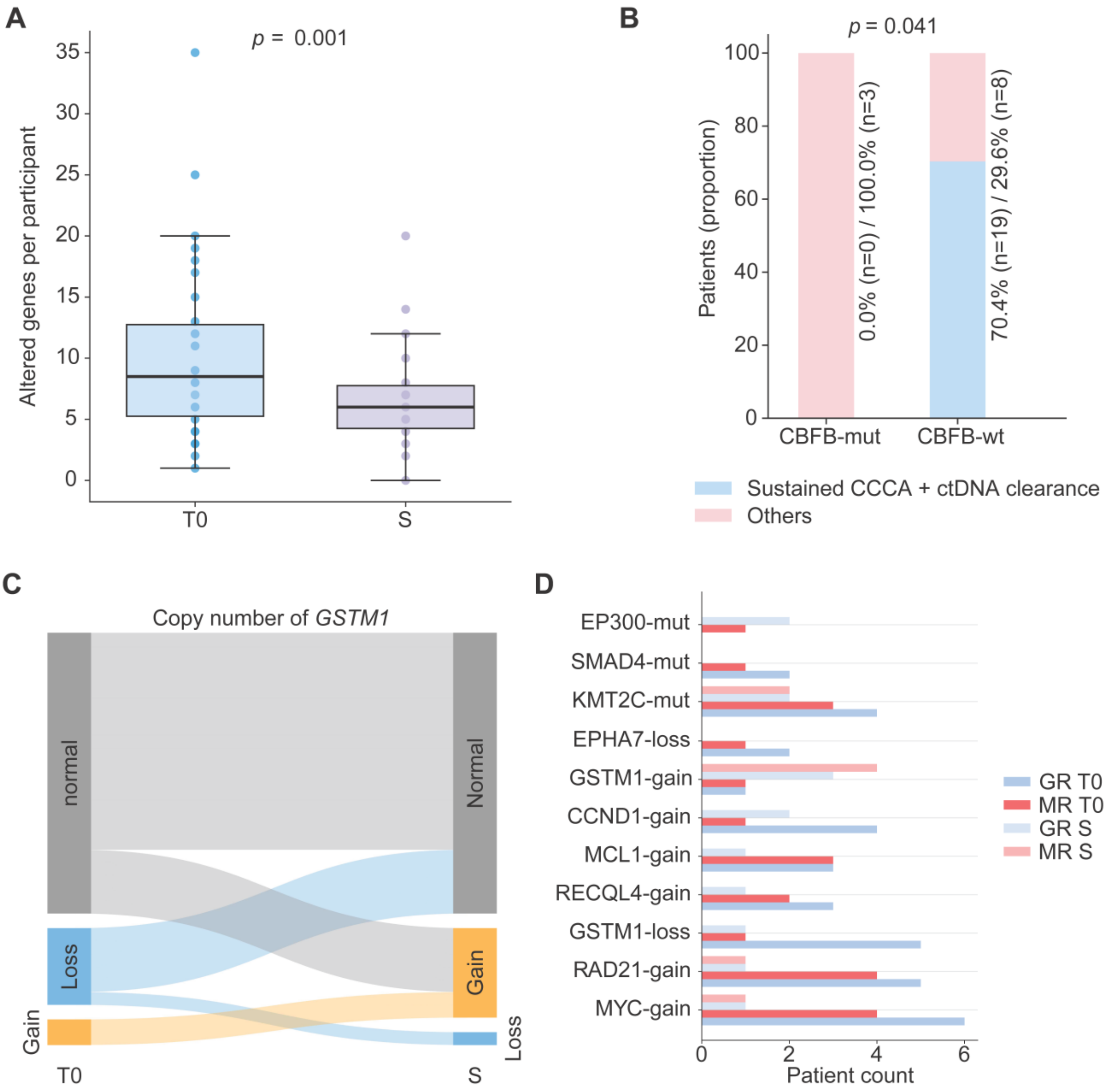

Figure S3. Dynamics of genetic variations after neoadjuvant therapy. Related to Figure 5.

(A) Significant decreases in variation counts in the overall population (n = 30) after neoadjuvant therapy.

(B) Wild-type *CBFB* was significantly correlated with sustained complete cell cycle arrest (CCCA) and concurrent circulating tumor DNA (ctDNA) clearance after neoadjuvant therapy.

(C) Sankey diagram illustrating dynamic changes in *GSTM1* copy numbers after neoadjuvant therapy. The copy number of *GSTM1* was categorized into deletion (loss), normal, and amplification (gain).

(D) Pattern of the top 11 most altered variant types after neoadjuvant therapy, grouped by treatment response (Good Responder [GR], n = 15; Moderate Responder [MR], n = 15).

T0: baseline; S: surgery; mut: mutation; wt: wild type.

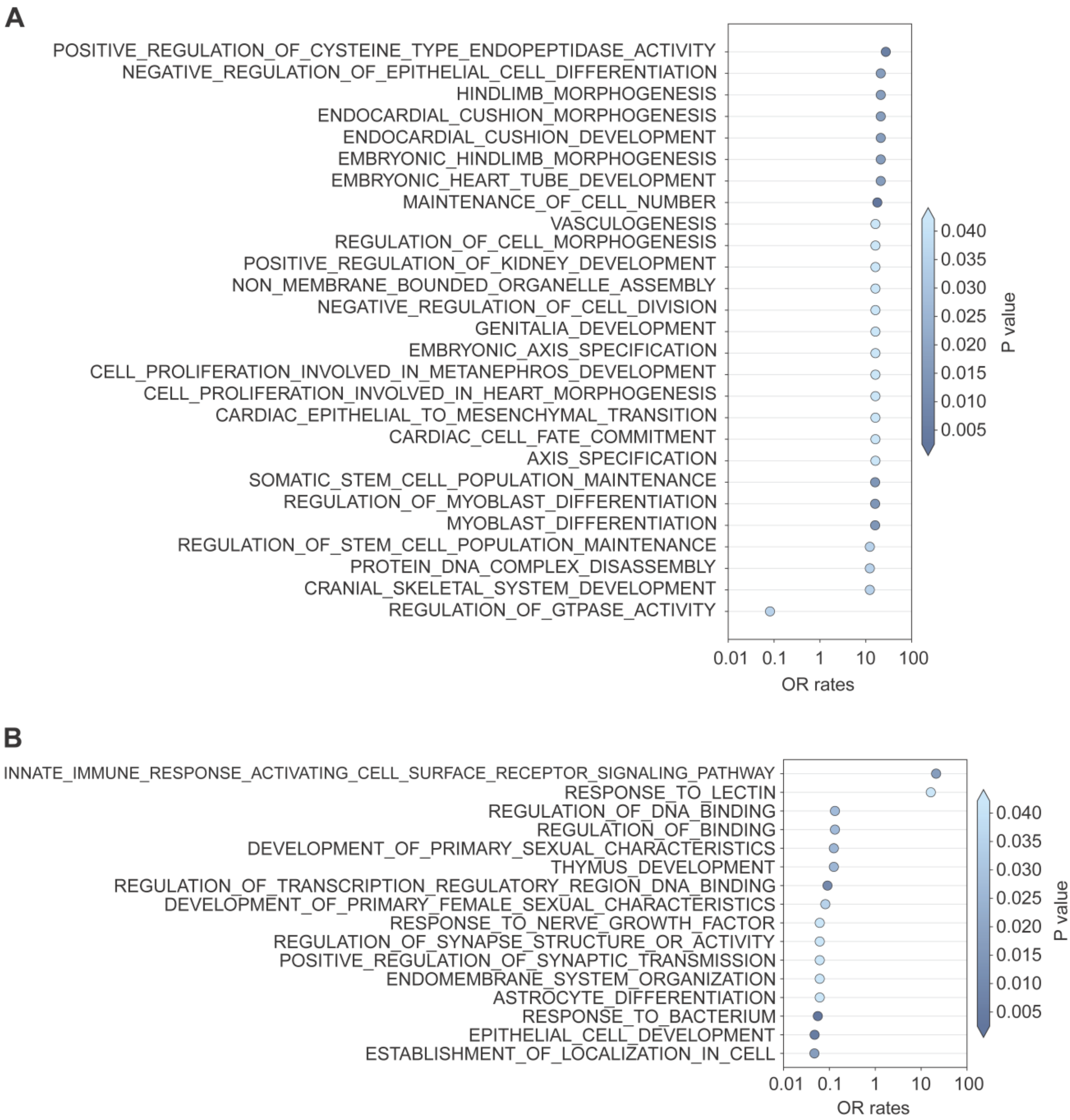

Figure S4. Longitudinal dynamics of pathway alterations during neoadjuvant therapy. Related to Figure 5.

(A) Analysis of alterations in gene sets derived from the Gene Ontology Biological Process ontology between Good Responders and Moderate Responders at 2 weeks post-dalpiciclib treatment (T1).

(B) Analysis of alterations in gene sets derived from the Gene Ontology Biological Process ontology between Good Responders and Moderate Responders at surgery (S).

OR: odds ratio.

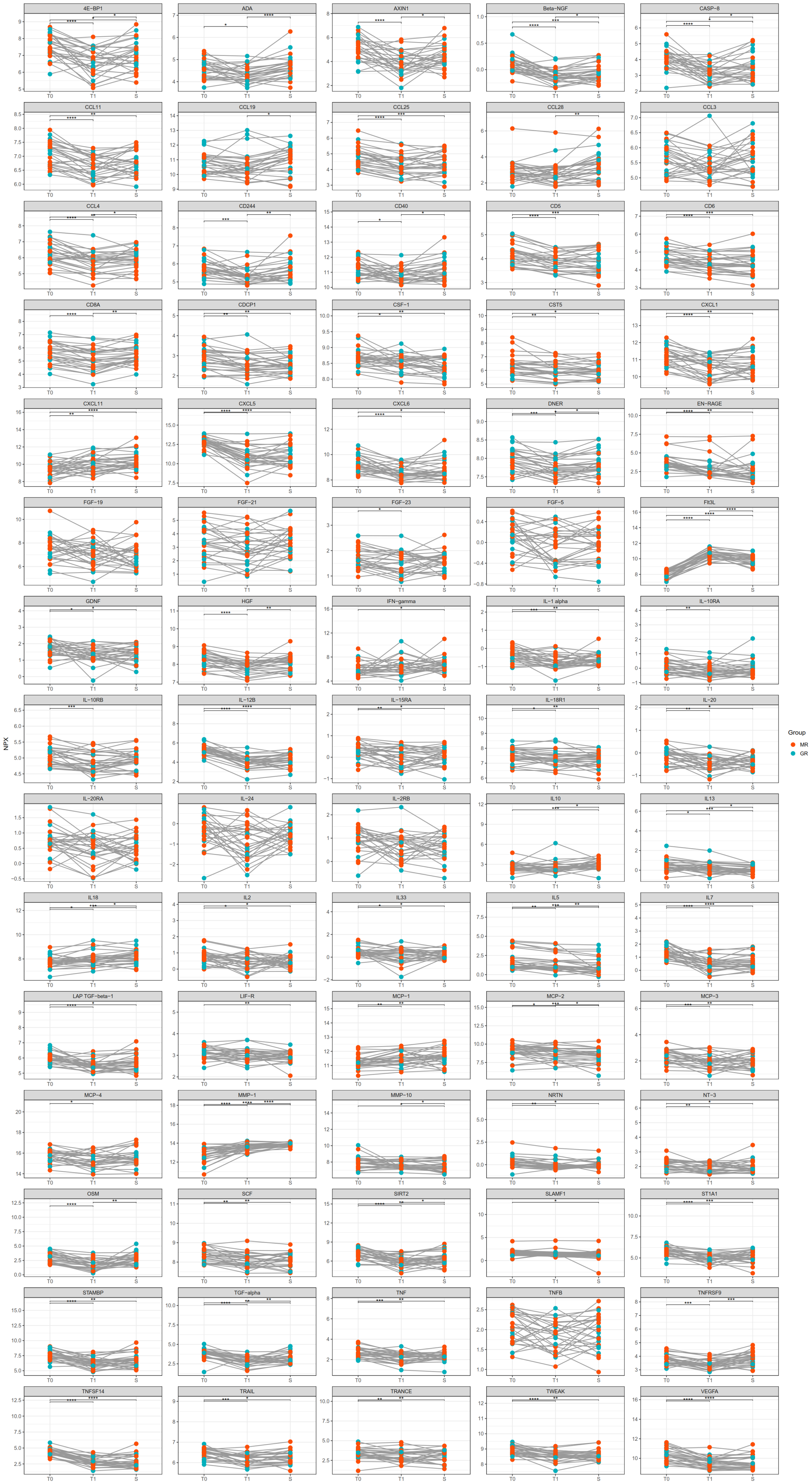
4E-BP1
ADA
AXIN1
Beta-NGF
CASP-8
CCL11
CCL19
CCL25
CCL28
CCL3
CCL4
CD244
CD40
CD5
CD6
CD8A
CDCP1
CSF-1
CST5
CXCL1
CXCL11
CXCL5
CXCL6
DNER
EN-RAGE
FGF-19
FGF-21
FGF-23
FGF-5
Flt3L
GDNF
HGF
IFN-gamma
IL-1 alpha
IL-10RA
IL-10RB
IL-12B
IL-15RA
IL-18R1
IL-20
IL-20RA
IL-24
IL-2RB
IL10
IL13
IL18
IL2
IL33
IL5
IL7
LAP TGF-beta-1
LIF-R
MCP-1
MCP-2
MCP-3
MCP-4
MMP-1
MMP-10
NRTN
NT-3
OSM
SCF
SIRT2
SLAMF1
ST1A1
STAMBP
TGF-alpha
TNF
TNFB
TNFRSF9
TNFSF14
TRAIL
TRANCE
TWEAK
VEGFA
NPX
T0
T1
S
Group
MR
GR

Figure S5. Dynamics of plasma proteins in 28 patients with matched samples at all three time points, showing significant changes during neoadjuvant therapy. Related to Figure 6.

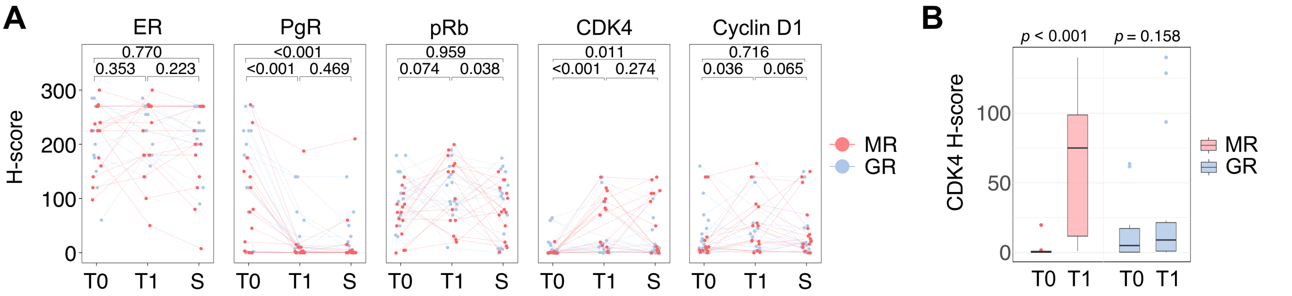

Figure S6. Dynamics of immunohistochemical biomarkers after neoadjuvant therapy. Related to Figure 6.

(A) H-scores of five immunohistochemical biomarkers across the time points in the overall population (*n* = 30).

(B) Expression levels of CDK4 at baseline (T0) and 2 weeks post-dalpiciclib treatment (T1) in patients with different treatment responses (Good Responder [GR], *n* = 15; Moderate Responder [MR], *n* = 15).

S: surgery; ER: estrogen receptor; PgR: progesterone receptor; pRb: phosphorylated retinoblastoma protein; CDK: cyclin-dependent kinase.

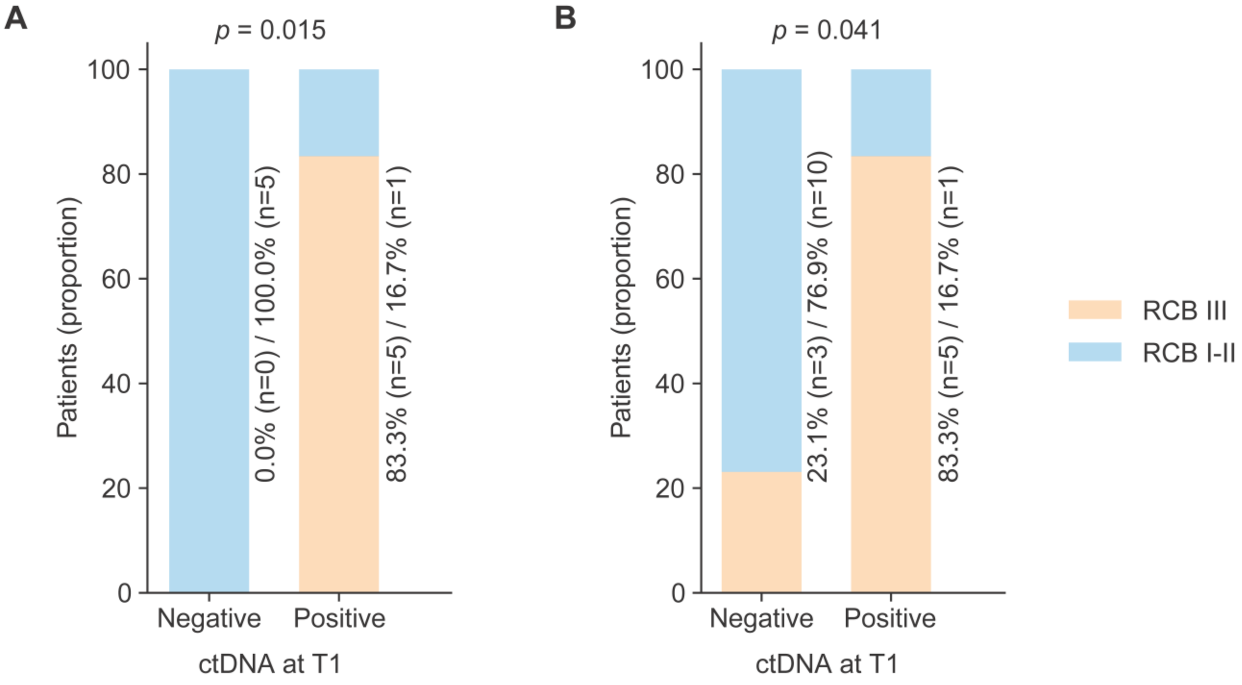

Figure S7. Prognostic value of circulating tumor DNA (ctDNA) positivity at 2 weeks post-dalpiciclib treatment (T1) in the Baseline Response Index (BRI)-high subgroup. Related to Figure 6.

(A) Comparison of residual cancer burden (RCB)-III proportions between patients with ctDNA clearance (T1-negative) and persistence (T1-positive) at T1 among BRI-high patients with ctDNA positivity detected at least once during monitoring (n = 11).

(B) RCB-III distribution by ctDNA status at T1 in the entire BRI-high subgroup (n = 19).

P-values were determined using Fisher's exact test.

Supplementary Tables

Table S1. Summary of common treatment-emergent adverse events (TEAEs).

**TEAEs ≥ 10%, *n* (%)**	**Dalpiciclib + anastrozole/letrozole (*n* = 30)**				
	**All grades**	**Grade 1**	**Grade 2**	**Grade 3**	**Grade 4**
Any adverse event	30 (100.0%)	0	10 (33.3%)	15 (50.0%)	5 (16.7%)
Neutropenia	29 (96.7%)	0	9 (30.0%)	16 (53.3%)	4 (13.3%)
Leukopenia	29 (96.7%)	3 (10.0%)	14 (46.7%)	11 (36.7%)	1 (3.3%)
Rash	13 (43.3%)	9 (30.0%)	4 (13.3%)	0	0
Pruritus	12 (40.0%)	8 (26.7%)	4 (13.3%)	0	0
Fatigue	12 (40.0%)	12 (40.0%)	0	0	0
Anemia	11 (36.7%)	9 (30.0%)	2 (6.7%)	0	0
Blood creatinine increased	11 (36.7%)	11 (36.7%)	0	0	0
Hot flush	11 (36.7%)	11 (36.7%)	0	0	0
Lymphopenia	9 (30.0%)	1 (3.3%)	7 (23.3%)	1 (3.3%)	0
Hypertriglyceridemia	9 (30.0%)	9 (30.0%)	0	0	0
Hyperglycemia	8 (26.7%)	8 (26.7%)	0	0	0
Constipation	8 (26.7%)	8 (26.7%)	0	0	0
Decreased appetite	7 (23.3%)	7 (23.3%)	0	0	0
Nausea	6 (20.0%)	6 (20.0%)	0	0	0
Stomatitis	6 (20.0%)	5 (16.7%)	0	1 (3.3%)	0
Thrombocytopenia	6 (20.0%)	5 (16.7%)	1 (3.3%)	0	0
Insomnia	6 (20.0%)	6 (20.0%)	0	0	0
Hyperphosphatemia	6 (20.0%)	6 (20.0%)	0	0	0
Arthralgia	6 (20.0%)	6 (20.0%)	0	0	0
Weight gain	5 (16.7%)	5 (16.7%)	0	0	0
Weight loss	4 (13.3%)	4 (13.3%)	0	0	0
Hyperuricaemia	4 (13.3%)	4 (13.3%)	0	0	0
Diarrhoea	4 (13.3%)	4 (13.3%)	0	0	0
Alopecia	4 (13.3%)	4 (13.3%)	0	0	0
Upper respiratory infection	3 (10.0%)	1 (3.3%)	2 (6.7%)		
Hypercholesterolemia	3 (10.0%)	3 (10.0%)	0	0	0
Electrocardiogram QTc prolonged	3 (10.0%)	3 (10.0%)	0	0	0

Note: For each patient, only the highest grade per adverse event term was recorded for severity assessment. No grade 5 adverse events were observed.

Table S2. Association between the timing of neutropenia occurrence and dalpiciclib dose reduction.

Subjects	First neutropenia event	First ≥ grade 3 neutropenia event	Dose reduction
P09	C1D7	C1D15	Yes
P13	C1D7	C1D15	Yes
P14	C1D7	C1D15	Yes
P26	C1D7	C1D28	Yes
P02	C1D15	C1D15	Yes
P08	C1D15	C1D15	Yes
P11	C1D15	C1D15	Yes
P21	C1D15	C1D15	Yes
P30	C1D15	C1D15	Yes
P15	C1D15	C1D28	Yes
P20	C1D7	C1D28	No
P05	C1D15	C1D15	No
P12	C1D15	C1D15	No
P18	C1D15	C1D15	No
P23	C1D15	C1D15	No
P25	C1D15	C1D28	No
P27	C1D15	C1D28	No
P07	C1D15	C2D28	No
P19	C1D15	C2D28	No
P06	C1D15	C4D21	No
P03	C1D15	NA	No
P04	C1D15	NA	No
P10	C1D15	NA	No
P16	C1D15	NA	No
P17	C1D15	NA	No
P22	C1D15	NA	No
P24	C1D15	NA	No
P28	C1D15	NA	No
P29	C1D15	NA	No
P01	NA	NA	No

Abbreviations: AE, adverse event; C, Cycle; D, Day; NA, not applicable.

Table S3. Baseline patient characteristics according to response status.

Characteristic	Good Responder (*n*, %)	Moderate Responder (*n*, %)	*p*-value
Age (years), median (range)	50.0 (38.0–73.0)	61.0 (38.0–76.0)	0.245
Menstrual status			0.715
Premenopausal	8 (53.3)	6 (40.0)	
Postmenopausal	7 (46.7)	9 (60.0)	
Clinical tumor stage			1.000
T2	9 (60.0)	9 (60.0)	
T3/4	6 (40.0)	6 (40.0)	
Clinical nodal stage			0.143
N0	2 (13.3)	3 (20.0)	
N1	12 (80.0)	7 (46.7)	
N2	1 (6.7)	5 (33.3)	
Clinical tumor stage			0.710
II	10 (66.7)	8 (53.3)	
III	5 (33.3)	7 (46.7)	
Histological type			0.732
IDC	14 (93.3)	12 (80.0)	
ILC	1 (6.7)	1 (6.7)	
Mixed*	0	2 (13.3)	
Histological tumor grade			0.483
1–2	13 (86.7)	15 (100.0)	
3	2 (13.3)	0	
ER expression			1.000
11–66%	3 (20.0)	2 (13.3)	
> 66%	12 (80.0)	13 (86.7)	
PgR expression			0.545
< 1%	1 (6.7)	3 (20.0)	
≥ 1%, < 20%	2 (13.3)	3 (20.0)	
≥ 20%	12 (80.0)	9 (60.0)	
Ki67 expression			0.224
< 20%	0	3 (20.0)	
≥ 20%	15 (100.0)	12 (80.0)	
MammaPrint/BluePrint signature			1.000
Luminal A	3 (20.0)	3 (20.0)	
Luminal B	11 (73.3)	12 (80.0)	
Basal	1 (6.7)	0	

*Including two cases of mixed carcinoma, each with 10–90% lobular or micropapillary subtype admixed with invasive ductal carcinoma (IDC).

Abbreviations: ILC, invasive lobular carcinoma; ER, estrogen receptor; PgR, progesterone receptor.

Table S4. Molecular Signatures Database (MSigDB) Gene Ontology gene sets related to response status ($p < 0.1$).

Gene Ontology (GO) Term	Contingency table	Odds ratio	*p*-value
GOBP_REGULATION_OF_INTRACELLULAR_ESTROGEN_RECEPTOR_SIGNALING_PATHWAY	[[7, 8], [0, 15]]	27.35294	0.006322
GOBP_FATTY_ACID_METABOLIC_PROCESS	[[11, 4], [3, 12]]	11	0.009221
GOBP_REGULATION_OF_TRANSCRIPTION_REGULATORY_REGION_DNA_BINDING	[[3, 12], [11, 4]]	0.090909	0.009221
GOBP_HINDLIMB_MORPHOGENESIS	[[8, 7], [1, 14]]	16	0.014193
GOBP_INTRACELLULAR_ESTROGEN_RECEPTOR_SIGNALING_PATHWAY	[[8, 7], [1, 14]]	16	0.014193
GOBP_DNA_STRAND_RESECTION_INVOLVED_IN_REPLICATION_FORK_PROCESSING	[[6, 9], [0, 15]]	21.21053	0.016858
GOBP_POSITIVE_REGULATION_OF_INTRACELLULAR_STEROID_HORMONE_RECEPTOR_ SIGNALING_PATHWAY	[[6, 9], [0, 15]]	21.21053	0.016858
GOBP_MONOCARBOXYLIC_ACID_BIOSYNTHETIC_PROCESS	[[10, 5], [3, 12]]	8	0.025328
GOBP_ORGANIC_ACID_BIOSYNTHETIC_PROCESS	[[10, 5], [3, 12]]	8	0.025328
GOBP_THYMUS_DEVELOPMENT	[[3, 12], [10, 5]]	0.125	0.025328
GOBP_HOMOLOGOUS_RECOMBINATION	[[11, 4], [4, 11]]	7.5625	0.026838
GOBP_NEURON_MIGRATION	[[4, 11], [11, 4]]	0.132231	0.026838
GOBP_REGULATION_OF_DNA_BINDING	[[4, 11], [11, 4]]	0.132231	0.026838
GOBP_SOMATIC_STEM_CELL_POPULATION_MAINTENANCE	[[11, 4], [4, 11]]	7.5625	0.026838
GOBP_CRANIAL_SKELETAL_SYSTEM_DEVELOPMENT	[[7, 8], [1, 14]]	12.25	0.035182
GOBP_EMBRYONIC_HINDLIMB_MORPHOGENESIS	[[7, 8], [1, 14]]	12.25	0.035182
GOBP_CELL_SURFACE_PATTERN_RECOGNITION_RECEPTOR_SIGNALING_PATHWAY	[[5, 10], [0, 15]]	16.2381	0.042146
GOBP_FORMATION_OF_EXTRACHROMOSOMAL_CIRCULAR_DNA	[[5, 10], [0, 15]]	16.2381	0.042146
GOBP_ESTABLISHMENT_OF_PROTEIN_LOCALIZATION	[[10, 5], [15, 0]]	0.061584	0.042146
GOBP_PROTEIN_TRANSPORT	[[10, 5], [15, 0]]	0.061584	0.042146
GOBP_MAINTENANCE_OF_LOCATION	[[8, 7], [2, 13]]	7.428571	0.050175
GOBP_POSITIVE_REGULATION_OF_DNA_REPAIR	[[8, 7], [2, 13]]	7.428571	0.050175
GOBP_SKELETAL_MUSCLE_ORGAN_DEVELOPMENT	[[2, 13], [8, 7]]	0.134615	0.050175
GOBP_MAINTENANCE_OF_CELL_NUMBER	[[12, 3], [6, 9]]	6	0.060433
GOBP_PROTEIN_POLYUBIQUITINATION	[[9, 6], [3, 12]]	6	0.060433
GOBP_REGULATION_OF_TRANSMEMBRANE_TRANSPORT	[[9, 6], [3, 12]]	6	0.060433
GOBP_ACTIVATION_OF_INNATE_IMMUNE_RESPONSE	[[10, 5], [4, 11]]	5.5	0.065595

GOBP_DNA_RECOMBINATION	[[11, 4], [5, 10]]	5.5	0.065595
GOBP_POSITIVE_REGULATION_OF_ANIMAL_ORGAN_MORPHOGENESIS	[[5, 10], [11, 4]]	0.181818	0.065595
GOBP_POSITIVE_REGULATION_OF_DEFENSE_RESPONSE	[[10, 5], [4, 11]]	5.5	0.065595
GOBP_RECOMBINATIONAL_REPAIR	[[10, 5], [4, 11]]	5.5	0.065595
GOBP_REGULATION_OF_BINDING	[[5, 10], [11, 4]]	0.181818	0.065595
GOBP_REGULATION_OF_MORPHOGENESIS_OF_AN_EPITHELIUM	[[5, 10], [11, 4]]	0.181818	0.065595
GOBP_SMALL_MOLECULE_BIOSYNTHETIC_PROCESS	[[10, 5], [4, 11]]	5.5	0.065595
GOBP_BONE_MORPHOGENESIS	[[6, 9], [1, 14]]	9.333333	0.080077
GOBP_NEGATIVE_REGULATION_OF_LIPID_BIOSYNTHETIC_PROCESS	[[6, 9], [1, 14]]	9.333333	0.080077
GOBP_ROOF_OF_MOUTH_DEVELOPMENT	[[6, 9], [1, 14]]	9.333333	0.080077
GOBP_ESTABLISHMENT_OF_LOCALIZATION_IN_CELL	[[11, 4], [15, 0]]	0.082437	0.099617
GOBP_NITROGEN_COMPOUND_TRANSPORT	[[11, 4], [15, 0]]	0.082437	0.099617
GOBP_CALCIUM_ION_TRANSMEMBRANE_TRANSPORT	[[4, 11], [0, 15]]	12.13043	0.099617
GOBP_CARDIAC_CELL_FATE_COMMITMENT	[[4, 11], [0, 15]]	12.13043	0.099617
GOBP_CELLULAR_RESPONSE_TO_GROWTH_HORMONE_STIMULUS	[[4, 11], [0, 15]]	12.13043	0.099617
GOBP_EPITHELIAL_CELL_MATURATION	[[4, 11], [0, 15]]	12.13043	0.099617
GOBP_POSITIVE_REGULATION_OF_INFLAMMATORY_RESPONSE	[[4, 11], [0, 15]]	12.13043	0.099617
GOMF_PROTEIN_SERINE_THREONINE_KINASE_ACTIVATOR_ACTIVITY	[[9, 6], [1, 14]]	21	0.005197
GOMF_AMINOACYLTRANSFERASE_ACTIVITY	[[6, 9], [0, 15]]	21.21053	0.016858
GOMF_KINASE_REGULATOR_ACTIVITY	[[15, 0], [10, 5]]	16.2381	0.042146
GOMF_CATALYTIC_ACTIVITY_ACTING_ON_A_NUCLEIC_ACID	[[9, 6], [3, 12]]	6	0.060433
GOMF_HYDROLASE_ACTIVITY_ACTING_ON_ACID_ANHYDRIDES	[[9, 6], [3, 12]]	6	0.060433
GOMF_TUBULIN_BINDING	[[9, 6], [3, 12]]	6	0.060433
GOMF_CHROMATIN_DNA_BINDING	[[1, 14], [6, 9]]	0.107143	0.080077
GOMF_FIBROBLAST_GROWTH_FACTOR_RECEPTOR_BINDING	[[4, 11], [0, 15]]	12.13043	0.099617
GOMF_NON_MEMBRANE_SPANNING_PROTEIN_TYROSINE_KINASE_ACTIVITY	[[4, 11], [0, 15]]	12.13043	0.099617
GOMF_OXIDOREDUCTASE_ACTIVITY	[[4, 11], [0, 15]]	12.13043	0.099617
GOMF_PHOSPHATIDYLINOSITOL_3_KINASE_ACTIVATOR_ACTIVITY	[[4, 11], [0, 15]]	12.13043	0.099617
GOCC_BRCA1_C_COMPLEX	[[6, 9], [0, 15]]	21.21053	0.016858

GOCC_NUCLEAR_CHROMOSOME	[[11, 4], [4, 11]]	7.5625	0.026838
GOCC_CELL_PROJECTION_MEMBRANE	[[5, 10], [0, 15]]	16.2381	0.042146
GOCC_CONDENSED_NUCLEAR_CHROMOSOME	[[10, 5], [4, 11]]	5.5	0.065595
GOCC_NUCLEAR_MEMBRANE	[[6, 9], [1, 14]]	9.333333	0.080077
GOCC_FIBRILLAR_CENTER	[[4, 11], [0, 15]]	12.13043	0.099617
GOCC_LEADING_EDGE_MEMBRANE	[[4, 11], [0, 15]]	12.13043	0.099617
GOCC_ORGANELLE_INNER_MEMBRANE	[[4, 11], [0, 15]]	12.13043	0.099617

Data in the contingency table are presented as [[The *n* of GR pts with pathway alterations, The *n* of GR pts without pathway alterations], [The *n* of MR pts with pathway alterations, The *n* of MR pts without pathway alterations]]. GR, Good Responder; MR, Moderate Responder; *n*, number; pts, patients.

Table S5. Activating events involving genes annotated to the *activation of innate immune response* gene set observed in Good Responders at baseline.

Patients	Gene	Mutation	Variation type
P01	LYN	/	Amplification
P03	PAK1*	/	Amplification
P09	NFKBIA	/	Amplification
P14	NFKBIA	/	Amplification
P15	LYN*	/	Amplification
P16	AKT1	c.49G>A, p.E17K	Activating mutation
P23	PAK1*	/	Amplification

*The activating event was also detected at surgery and involved in the gene set *innate immune response activating cell surface receptor signaling pathway*.

Table S6. The list of 769 genes included in the NGS panel.

ABCA13	CACNA2D1	DPP6	GNAQ	MAP3K1	PARD6B	RPF2	TNIK
ABCA8	CALD1	DPYD	GNAS	MAP3K13	PARK2	RPRD1A	TNKS
ABCB1	CALM2	DSCAM	GPAT3	MAP3K4	PARP1	RPS6KB1	TNRC1 8
ABCC2	CALR	E2F3	GPC4	MAP4K3	PARP2	RPTOR	TOP1
ABCC9	CARD11	EBP	GPM6A	MAP4K5	PARP3	RRM1	TOP2B
ABL1	CASP8	EED	GRB10	MAPK1	PARP8	RRP1B	TP53
ACADSB	CAST	EGFR	GREM1	MAPKAP1	PAX3	RUNX1	TP63
ACOT13	CBFB	EIF1AX	GRIK2	MAPKBP1	PAX5	RWDD1	TPH1
ACRC	CBL	EIF4E	GRIN2A	MARK1	PBRM1	RYBP	TPM1
ADCY8	CBR3	EIF4G3	GSK3B	MARK3	PDCD1	RYR2	TRA2A
ADGRG6	CBR4	ELFN1	GSKIP	MAX	PDCD1L G2	SASH1	TRAF7
AGAP1	CCDC157	ELMOD2	GSTA1	MCL1	PDE4D	SCOC	TRIM24
AK7	CCDC18	EML4	GSTM1	MDC1	PDGFRA	SDHA	TRIM25
AKT1	CCND1	ENOSF1	GSTP1	MDM2	PDGFRB	SDHAF2	TSC1
AKT2	CCND2	ENSA	GUCY1 A2	MDM4	PDPK1	SDHB	TSC2
AKT3	CCND3	EP300	H3F3A	MED12	PDS5A	SDHC	TSHR
ALDH5A1	CCNE1	EPCAM	HAUS2	MED12L	PFKP	SDHD	TSN
ALG9	CD274	EPG5	HAUS6	MED14	PGBD1	SEL1L3	TTC1
ALK	CD40	EPHA3	HCAR2	MED19	PGR	SEMA3C	TTC6
ALOX12B	CD74	EPHA5	HDGFR P3	MEF2BNB-MEF2B	PGRMC2	SEMA3E	TTN
ALS2CR11	CD79A	EPHA7	HERC6	MEIS1	PHF20	SERTAD4	TUBD1
AMBRA1	CD79B	EPHB1	HEY1	MEN1	PIGF	SETD2	TXNDC 16
AMER1	CDA	EPYC	HGF	MET	PIK3C2G	SF3B1	TXNRD 1
ANAPC7	CDC73	ERBB2	HIST1H 1C	METTL9	PIK3C3	SFXN4	U2AF1
ANKRD28	CDCA8	ERBB3	HIST1H 3B	MITF	PIK3CA	SH2D1A	UBAP2 L
ANKRD46	CDH1	ERBB4	HLA-A	MLH1	PIK3CB	SHQ1	UBE2E3
ANO1	CDK12	ERCC1	HLA-B	MLH3	PIK3CD	SHROOM3	UBE4A
APAF1	CDK4	ERCC2	HLA-C	MMP16	PIK3CG	SIMC1	UBN2
APC	CDK6	ERCC3	HMCN1	MMP3	PIK3R1	SIPA1L2	UBXN7
APOL2	CDK8	ERCC4	HNF1A	MPL	PIK3R2	SKA3	UGT1A 1
APOPT1	CDKL3	ERG	HNF4A	MRE11A	PIK3R3	SLC13A1	ULK2
AQR	CDKN1A	ERI1	HOMER 1	MRPL19	PIM1	SLC22A2	ULK4
AR	CDKN1B	ERRFI1	HRAS	MS4A13	PKHD1	SLC25A13	UMPS
ARAF	CDKN2A	ESR1	HSD17B 11	MSANTD3-TMEFF1	PLCG2	SLC30A5	UPF2
ARHGAP2 6	CDKN2B	ETV1	HSD3B1	MSH2	PLEKHA 1	SLC31A1	USP11
ARHGAP4	CDKN2C	ETV4	HSPA1B	MSH3	PLEKHH 2	SLC35B1	USP34
ARHGAP6	CDO1	ETV5	HSPA4	MSH6	PLXNC1	SLC7A8	USP9Y
ARHGEF1 2	CEBPA	ETV6	HSPA5	MTF1	PMS1	SLC9C2	UTS2
ARHGEF3	CEP120	EWSR1	HSPH1	MTF2	PMS2	SLCO1B1	UTY
ARID1A	CEP290	EXOSC8	HTT	MTHFR	PNO1	SLCO1B3	VEGFA
ARID1B	CFAP221	EZH2	HYOU1	MTOR	POLA1	SLIT1	VHL
ARID2	CFAP53	EZR	IARS	MTR	POLD1	SLX4	VSIG10
ARID4A	CHD1	FAM149A	ICOSLG	MTRR	POLE	SMAD2	WDR5
ARID5B	CHD2	FAM153B	ID2	MUTYH	POSTN	SMAD3	WHSC1

ARL13B	CHEK1	FAM161A	ID3	MYADM	PPARG	SMAD4	WHSC1L1
ARL4A	CHEK2	FAM175A	IDH1	MYB	PPP1R21	SMARCA4	WT1
ARL6IP6	CHRM3	FAM184B	IDH2	MYC	PPP2R1A	SMARCB1	XIAP
ARMC5	CHURC1-FNTB	FAM20A	IGF1	MYCL	PRDM1	SMO	XPC
ASB11	CIC	FAM46C	IGF1R	MYCN	PRELID3B	SNX6	XPO1
ASH1L	CLASP2	FANCA	IGF2	MYD88	PREX2	SOCS1	XRCC1
ASPH	CLEC16A	FANCC	IKBKE	MYO10	PRKAR1A	SOD2	XRCC2
ASXL1	CLEC9A	FANCD2	IKZF1	MYOD1	PRKCI	SOX17	YAP1
ASXL2	CNKSR3	FANCF	IL10	MYOM1	PRKDC	SOX2	YLPM1
ATG3	CNOT8	FANCG	IL13RA1	MZT2A	PRPF39	SOX9	YWHAE
ATG4C	COL15A1	FAS	IL7R	NAB1	PRPF4	SPEN	ZBBX
ATIC	COX18	FAT1	IMPG1	NAMPT	PTCH1	SPOP	ZBTB40
ATM	CPS1	FBXO11	INHBA	NAPG	PTEN	SRC	ZDHHC17
ATP6V0A1	CREBBP	FBXW7	INPP4A	NAV1	PTK2	SRSF3	ZDHHC20
ATP6V0A2	CRKL	FGF10	INPP4B	NBAS	PTPN11	SRY	ZMYM2
ATP6V0A4	CRLF2	FGF16	IRF4	NBEAL1	PTPN4	STAB2	ZMYM4
ATP6V0E1	CSF1R	FGF19	IRF6	NBN	PTPRD	STAG2	ZNF195
ATP8A1	CSF3R	FGF3	IRF8	NCOA6	PTPRJ	STARD4	ZNF2
ATR	CTAGE5	FGF4	IRS2	NCOR1	PTPRS	STAT3	ZNF280D
ATRX	CTCF	FGF6	ITGAL	NEDD4L	PTPRT	STK11	ZNF283
AURKA	CTLA4	FGFR1	JAK1	NEO1	PURA	STMN1	ZNF367
AURKB	CTNNB1	FGFR2	JAK2	NF1	RAB2B	STRBP	ZNF711
AXIN1	CTSC	FGFR3	JAK3	NF2	RABGAP1L	STT3A	ZNF805
AXIN2	CUL3	FGFR4	JUN	NFE2L2	RAC1	STYX	ZNF91
AXL	CXCL8	FH	KDM5A	NFKBIA	RAD21	SUCLG1	ZZZ3
B2M	CXCR4	FLCN	KDM5C	NFXL1	RAD50	SUFU	
BAP1	CYBA	FLI1	KDM6A	NKAP	RAD51	SUGCT	
BARD1	CYFIP1	FLOT1	KDR	NKX2-1	RAD51B	SUZ12	
BCAS1	CYLD	FLT1	KEAP1	NLRP7	RAD51C	SYK	
BCL2	CYP19A1	FLT3	KIAA1210	NOTCH1	RAD51D	SYNE2	
BCL2L1	CYP2B6	FLT4	KIAA1841	NOTCH2	RAD52	TAF15	
BCL2L11	CYP2C19	FMNL2	KIT	NOTCH3	RAD54L	TAOK3	
BCL6	CYP2C8	FMO1	KLF4	NOTCH4	RAF1	TARBP1	
BCOR	CYP2D6	FMR1	KMT2A	NPM1	RALGAPB	TBC1D8B	
BCR	DARS2	FNBP4	KMT2C	NR1I3	RAP2B	TBCD	
BIRC3	DAXX	FOLH1B	KMT2D	NRAS	RARA	TBX3	
BIVM-ERCC5	DCHS2	FOXA1	KPNA4	NRG1	RASA1	TECPR2	
BLM	DDR1	FOXL2	KPNB1	NRG4	RB1	TENM3	
BMPR1A	DDR2	FOXO1	KRAS	NSD1	RBM10	TERT	
BRAF	DDX19B	FOXP1	KTN1	NT5C2	RBM27	TERT-promoter	
BRCA1	DDX58	FPGT-TNNI3K	LAMA3	NTHL1	RECQL4	TET1	
BRCA2	DEPDC5	FUBP1	LATS1	NTRK1	REL	TET2	
BRD4	DHFR	FUS	LATS2	NTRK2	RET	TFDP1	
BRIP1	DIAPH1	FXR1	LEPR	NTRK3	RFC1	TFRC	

BRMS1L	DIAPH2	GABRP	LMO1	NUDT13	RFWD2	TGFBR1	
BRS3	DICER1	GALNT12	LNPEP	NUP85	RHOA	TGFBR2	
BTF3	DIS3	GALNT14	LONRF3	NUP93	RHOT1	TMEM126B	
BTG1	DLC1	GANC	LRP2	OSBP	RIC1	TMEM127	
BTK	DMXL1	GATA1	LRRC16A	OTOGL	RICTOR	TMEM132D	
C22orf23	DNAJB1	GATA2	LRRC34	OTOS	RIPK2	TMEM67	
C5orf15	DNAJC11	GATA3	LYN	P2RY8	RIT1	TMPRSS15	
C5orf42	DNMT1	GIPC1	MALRD1	PAK1	RNF112	TMPRSS2	
C7orf66	DNMT3A	GLI1	MALT1	PAK7	RNF19A	TMTC4	
C8orf34	DNMT3B	GMEB1	MAP2K1	PALB2	RNF43	TNFAIP3	
CAB39	DOCK11	GNA11	MAP2K2	PAPOLG	ROBO1	TNFRSF14	
CACNA1E	DOT1L	GNA13	MAP2K4	PAQR8	ROS1	TNFSF13B	

Table S7. The list of 92 proteins included in Olink Target 96 inflammation panel.

Uniprot ID	Protein	Gene name
O00300	Osteoprotegerin (OPG)	TNFRSF11B
O14625	C-X-C motif chemokine 11 (CXCL11)	CXCL11
O14788	TNF-related activation-induced cytokine (TRANCE)	TNFSF11
O15169	Axin-1 (AXIN1)	AXIN1
O15444	C-C motif chemokine 25 (CCL25)	CCL25
O43508	Tumor necrosis factor (Ligand) superfamily, member 12 (TWEAK)	TNFSF12
O43557	Tumor necrosis factor ligand superfamily member 14 (TNFSF14)	TNFSF14
O95630	STAM-binding protein (STAMPB)	STAMBP
O95750	Fibroblast growth factor 19 (FGF-19)	FGF19
O95760	Interleukin-33 (IL-33)	IL33
P00749	Urokinase-type plasminogen activator (uPA)	PLAU
P00813	Adenosine Deaminase (ADA)	ADA
P01135	Transforming growth factor alpha (TGF-alpha)	TGFA
P01137	Latency-associated peptide transforming growth factor beta-1 (LAP TGF-beta-1)	TGFB1
P01138	Beta-nerve growth factor (Beta-NGF)	NGF
P01374	TNF-beta (TNFB)	LTA
P01375	Tumor necrosis factor (TNF)	TNF
P01579	Interferon gamma (IFN-gamma)	IFNG
P01583	Interleukin-1 alpha (IL-1 alpha)	IL1A
P01732	T-cell surface glycoprotein CD8 alpha chain (CD8A)	CD8A
P02778	C-X-C motif chemokine 10 (CXCL10)	CXCL10
P03956	Matrix metalloproteinase-1 (MMP-1)	MMP1
P05112	Interleukin-4 (IL-4)	IL4
P05113	Interleukin-5 (IL5)	IL5
P05231	Interleukin-6 (IL6)	IL6
P06127	T-cell surface glycoprotein CD5 (CD5)	CD5
P09238	Matrix metalloproteinase-10 (MMP-10)	MMP10
P09341	C-X-C motif chemokine 1 (CXCL1)	CXCL1
P09603	Macrophage colony-stimulating factor 1 (CSF-1)	CSF1
P10145	Interleukin-8 (IL-8)	CXCL8
P10147	C-C motif chemokine 3 (CCL3)	CCL3
P12034	Fibroblast growth factor 5 (FGF-5)	FGF5
P13232	Interleukin-7 (IL-7)	IL7
P13236	C-C motif chemokine 4 (CCL4)	CCL4
P13500	Monocyte chemotactic protein 1 (MCP-1)	CCL2
P13725	Oncostatin-M (OSM)	OSM
P14210	Hepatocyte growth factor (HGF)	HGF
P14784	Interleukin-2 receptor subunit beta (IL-2RB)	IL2RB
P15018	Leukemia inhibitory factor (LIF)	LIF
P15692	Vascular endothelial growth factor A (VEGF-A)	VEGFA
P20783	NEUtrophin-3 (NT-3)	NTF3
P21583	Stem cell factor (SCF)	KITLG
P22301	Interleukin-10 (IL10)	IL10
P25942	CD40L receptor (CD40)	CD40
P28325	Cystatin D (CST5)	CST5
P29460	Interleukin-12 subunit beta (IL-12B)	IL12B

P30203	T cell surface glycoprotein CD6 isoform (CD6)	CD6
P35225	Interleukin-13 (IL-13)	IL13
P39905	Glial cell line-derived NEUtrophic factor (GDNF)	GDNF
P42702	Leukemia inhibitory factor receptor (LIF-R)	LIFR
P42830	C-X-C motif chemokine 5 (CXCL5)	CXCL5
P49771	Fms-related tyrosine kinase 3 ligand (Flt3L)	FLT3LG
P50225	Sulfotransferase 1A1 (ST1A1)	SULT1A1
P50591	TNF-related apoptosis-inducing ligand (TRAIL)	TNFSF10
P51671	Eotaxin (CCL11)	CCL11
P55773	C-C motif chemokine 23 (CCL23)	CCL23
P60568	Interleukin-2 (IL-2)	IL2
P78423	Fractalkine (CX3CL1)	CX3CL1
P78556	C-C motif chemokine 20 (CCL20)	CCL20
P80075	Monocyte chemotactic protein 2 (MCP-2)	CCL8
P80098	Monocyte chemotactic protein 3 (MCP-3)	CCL7
P80162	C-X-C motif chemokine 6 (CXCL6)	CXCL6
P80511	Protein S100-A12 (EN-RAGE)	S100A12
Q07011	Tumor necrosis factor receptor superfamily member 9 (TNFRSF9)	TNFRSF9
Q07325	C-X-C motif chemokine 9 (CXCL9)	CXCL9
Q08334	Interleukin-10 receptor subunit beta (IL-10RB)	IL10RB
Q13007	Interleukin-24 (IL-24)	IL24
Q13261	Interleukin-15 receptor subunit alpha (IL-15RA)	IL15RA
Q13291	Signaling lymphocytic activation molecule (SLAMF1)	SLAMF1
Q13478	Interleukin-18 receptor 1 (IL-18R1)	IL18R1
Q13541	Eukaryotic translation initiation factor 4E-binding protein 1 (4E-BP1)	EIF4EBP1
Q13651	Interleukin-10 receptor subunit alpha (IL-10RA)	IL10RA
Q14116	Interleukin-18 (IL-18)	IL18
Q14790	Caspase-8 (CASP-8)	CASP8
Q16552	Interleukin-17A (IL-17A)	IL17A
Q5T4W7	Artemin (ARTN)	ARTN
Q8IXJ6	SIR2-like protein 2 (SIRT2)	SIRT2
Q8N6P7	Interleukin-22 receptor subunit alpha-1 (IL-22 RA1)	IL22RA1
Q8NFT8	Delta and Notch-like epidermal growth factor-related receptor (DNER)	DNER
Q969D9	Thymic stromal lymphopoietin (TSLP)	TSLP
Q99616	Monocyte chemotactic protein 4 (MCP-4)	CCL13
Q99731	C-C motif chemokine 19 (CCL19)	CCL19
Q99748	Neurturin (NRTN)	NRTN
Q9BZW8	Natural killer cell receptor 2B4 (CD244)	CD244
Q9GZV9	Fibroblast growth factor 23 (FGF-23)	FGF23
Q9H5V8	CUB domain-containing protein 1 (CDCP1)	CDCP1
Q9NRJ3	C-C motif chemokine 28 (CCL28)	CCL28
Q9NSA1	Fibroblast growth factor 21 (FGF-21)	FGF21
Q9NYY1	Interleukin-20 (IL-20)	IL20
Q9NZQ7	Programmed cell death 1 ligand 1 (PD-L1)	CD274
Q9P0M4	Interleukin-17C (IL-17C)	IL17C
Q9UHF4	Interleukin-20 receptor subunit alpha (IL-20RA)	IL20RA
